# Three-dimensional nanofibrous sponges with aligned architecture and controlled hierarchy regulate neural stem cell fate for spinal cord regeneration

**DOI:** 10.7150/thno.87288

**Published:** 2023-08-28

**Authors:** Zhiwei Li, Ye Qi, Lei Sun, Zheng Li, Shaojuan Chen, Yuqi Zhang, Yuan Ma, Jinming Han, Zide Wang, Yulin Zhang, Huimin Geng, Bin Huang, Jian Wang, Gang Li, Xingang Li, Shaohua Wu, Shilei Ni

**Affiliations:** 1 Department of Neurosurgery, Qilu Hospital of Shandong University and Institute of Brain and Brain-Inspired Science, Cheeloo College of Medicine, Shandong University, Jinan, Shandong, 250012, China.; 2 College of Textiles & Clothing, Qingdao University, Qingdao, China.; 3 Department of Endocrinology, Qilu Hospital of Shandong University and Institute of Endocrine and Metabolic Diseases of Shandong University, Jinan, Shandong, 250012, China.; 4 Department of Biomedicine, University of Bergen, Jonas Lies vei 91, 5009 Bergen, Norway.

**Keywords:** Nanofibrous sponge, Spinal cord injury, Electrospinning, Neural stem cells, Neural tissue engineering.

## Abstract

**Background:** Spinal cord injury (SCI) induces neuronal death and disrupts the nerve fiber bundles, which leads to severe neurological dysfunction and even permanent paralysis. A strategy combining biomimetic nanomaterial scaffolds with neural stem cell (NSC) transplantation holds promise for SCI treatment.

**Methods:** Innovative three-dimensional (3D) nanofibrous sponges (NSs) were designed and developed by a combination of directional electrospinning and subsequent gas-foaming treatment. Immunofluorescence, mRNA sequencing, magnetic resonance imaging, electrophysiological analysis, and behavioral tests were used to investigate the* in vitro* and *in vivo* regenerative effects of the 3D NSs.

**Results:** The generated 3D NSs exhibited uniaxially aligned nano-architecture and highly controllable hierarchical structure with super-high porosity (99%), outstanding hydrophilicity, and reasonable mechanical performance. They facilitated cell infiltration, induced cell alignment, promoted neuronal differentiation of NSCs, and enhanced their maturation mediated through cellular adhesion molecule pathways. *In vivo*, the NSC-seeded 3D NSs efficiently promoted axon reinnervation and remyelination in a rat SCI model, with new “neural relays” developing across the lesion gap. These histological changes were associated with regain of function, including increasing the neurological motor scores of SCI rats, from approximately 2 to 16 (out of 21), and decreasing the sensing time in the tape test from 140 s to 36 s. Additionally, the scaffolds led to restoration of ascending and descending electrophysiological signalling.

**Conclusion:** The as-fabricated 3D NSs effectively regulate NSC fates, and an advanced combination of 3D NS design and transplanted NSCs enables their use as an ideal tissue-engineered scaffold for SCI repair.

## Introduction

Over 500,000 patients worldwide suffer permanent deficits in sensory and motor function resulting from spinal cord injury (SCI), and the inability to recover from SCI generates a tremendous socio-economic burden 1. Unfortunately, current clinical treatments are only effective for SCI during the acute phase, and no therapeutic strategy is currently available to promote neural regeneration and reconstruct neural networks, due to the inhibitory and non-permissive microenvironment of SCI 2, 3. Neural tissue engineering (NTE) has recently emerged as a promising alternative for SCI repair. It has been shown to provide an instructive microenvironment to bridge the lesion gap of ascending and descending spinal tracts and support axon regeneration and functional recovery 4, 5.

A critical issue for NTE is the biomaterial scaffolds, which are designed to resemble the native extracellular matrix (ECM) and thus effectively promote the regeneration and repair of damaged neural tissues 6. In the past two decades, the design and construction of biomimetic nanomaterial scaffolds with high specific surface area, high porosity, and nano-architecture mimicking native ECM fibrils have attracted significant interest in the field of NTE. Although numerous strategies exist, including phase separation, self-assembly, and super-drawing, for the generation of nanomaterial-based scaffolds, electrospinning is a more promising approach for manufacturing nanofibrous scaffolds, because of its simplicity, versatility, and low cost 7, 8. The physical cues provided by electrospun nanofibers have been extensively demonstrated to improve cell-scaffold interactions, ECM deposition and remodelling, and even guide stem cell differentiation 9-11. Compared with chaotically oriented nanofiber scaffolds, nanofibers with aligned structures exhibit clear advantages for SCI repair. These advantages have been shown to effectively regulate the adhesion, elongation, orientation, and migration of neurons and glial cells, as well as effectively guide the directional regrowth and regeneration of axons at the SCI lesion site through the restoration of ascending and descending neural pathways and physiological function 12, 13. Unfortunately, most electrospun nanofibers are collected in the form of mat-like structures with two-dimensional (2D) dense structure and small pore sizes. Such features have inevitably yielded low cell infiltration and unsatisfactory regeneration outcomes for three-dimensional (3D) neural tissues 14, 15. Thus, to design and develop a novel electrospinning-based biomaterial scaffold integrated with uniaxially aligned structure and 3D ECM-mimicking hierarchical structure while maintaining the desirable nanofibrous characteristics and appropriate physicochemical properties remains a tremendous technical challenge.

Cells are also of significant importance for NTE applications 16. Neural stem cells (NSCs) retain self-renewal and multilineage differentiation abilities. In response to SCI, a few endogenous NSCs can be quickly activated and differentiated into different cell types 17. However, due to the harsh SCI microenvironment, most activated NSCs differentiate into astrocytes rather than neuronal lineage cells, making it difficult for them to take on neurological functions 18, 19. The implantation of exogenous propagated NSCs is considered a promising strategy for SCI repair 11. However, due to the lack of a support system, direct injection of NSCs into the lesion cavity does not achieve satisfactory results 20. Therefore, constructing an ideal cell scaffold that can effectively regulate NSC fate remains an intractable challenge for SCI treatment.

**Figure 1:**
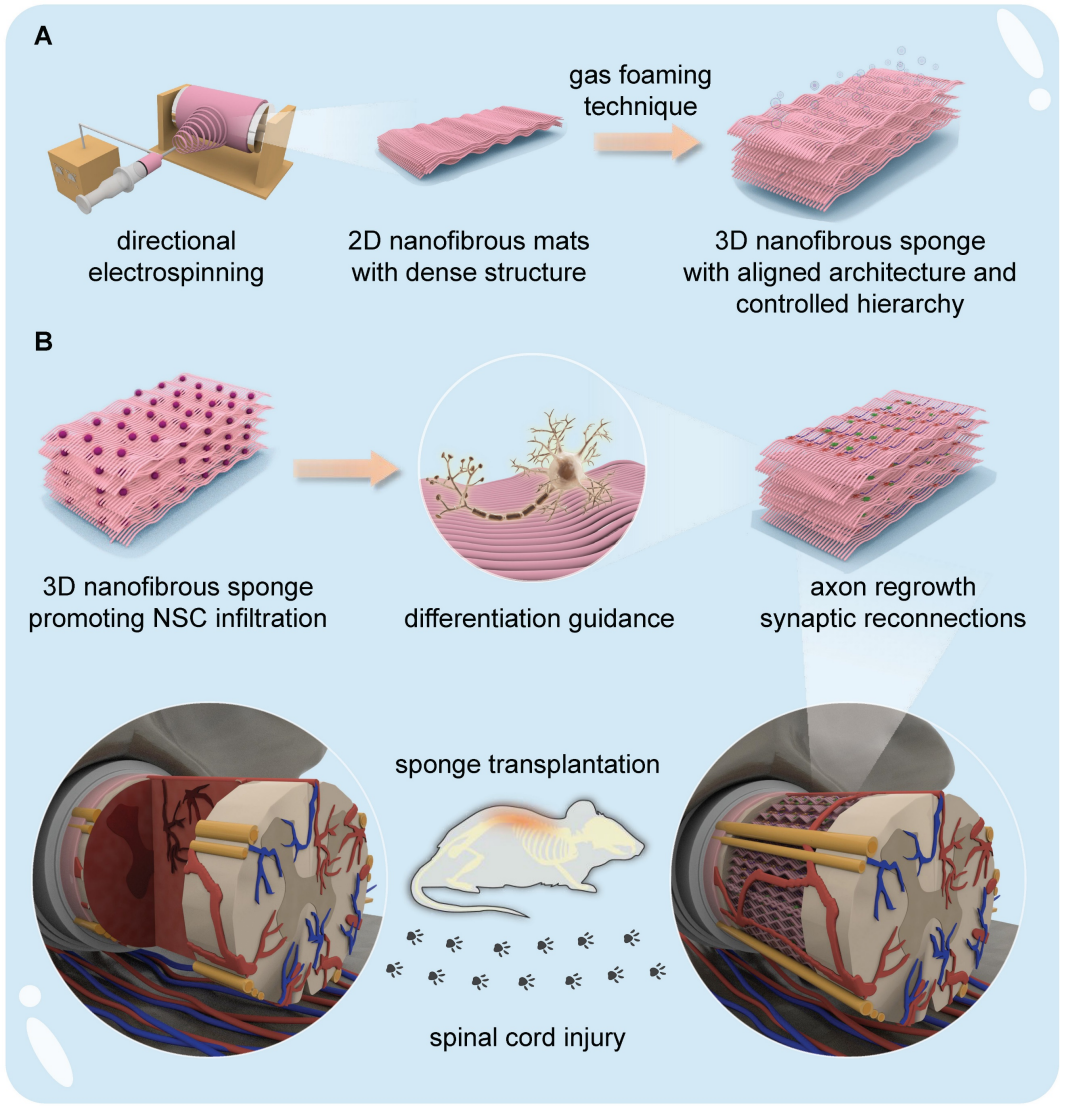
**Illustration of the fabrication of 3D NSs and the application of NSC-seeded 3D NSs for SCI treatment. (A)** A directional electrospinning method was performed to produce aligned 2D PCL/PPDO nanofibrous mats (NMs), which were subsequently expanded into 3D PCL/PPDO NSs through a gas-foaming technique. **(B)** Exogenous NSCs harvested from foetal rats were seeded onto the as-obtained 3D NSs, and the 3D NSs effectively regulated the differentiation fate of NSCs *in vitro*. Implantation of the NSC-NS constructs into the lesion gap served to bridge nerve stumps in an *in vivo* SCI rat model.

To overcome these challenges, we developed a universal strategy to transform the nanofibrous scaffolds from 2D to 3D, thus promoting neurogenesis after adult SCI. Specifically, an integrated strategy combining directional electrospinning with gas-foaming technology was used to generate 3D nanofibrous sponges (NSs) through gas bubble expansion between the adjacent nanofiber layers of electrospun 2D polycaprolactone (PCL)/poly(p-dioxanone) (PPDO) mats. The as-generated 3D NSs were investigated *in vitro* and *in vivo* as potential “cytosponges”, possessing a laminated structure with ECM-mimicking aligned nanotopography, controllable hierarchical structure, high porosity and hydrophilicity. The sponges thereby provided an instructive microenvironment for guiding neuronal differentiation and encouraging interaction between endogenous and exogenous cells. The internal spacing between the layers of 3D NSs was designed at a microscale level, which supported optimal widths for NSC infiltration and proliferation. Meanwhile, the aligned nanofibrous structure maintained in each layer guided the migration and alignment of both grafted and host neural cells. The *in vivo* regenerative effects of implantation of NSC-loaded 3D NSs were demonstrated in a rat model for SCI. A schematic illustration of the study is shown in **Figure 1**.

## Methods

### Ethics statement

Animal experiments were approved by the Animal Care and Experiment Committee of Qilu Hospital affiliated with Shandong University (approval No.: DWLL-2021-005) and carried out following the local animal care guidelines.

### Preparation of 2D PCL/PPDO NMs

PCL (MW = 80,000; Sigma Aldrich; St. Louis, IL, USA) and PPDO (MW = 100,000; Corbion; Purac, Netherlands) with a mass ratio of 4:1 were dissolved in hexafluoro-2-propanol (HFIP, purity ≥ 99.8%; Aladdin Reagent; Shanghai, China) to generate a homogeneous spinning solution with a total concentration of 10% (w/v). A directional electrospinning device employing a rotating cylinder as a nanofiber collector was used to spin the PCL/PPDO solution into uniaxially aligned nanofibers. The applied voltage, spinning distance, and solution feeding rate were set at 12 kV, 16 cm, and 0.8 mL/h, respectively. The rotating speed of the cylinder collector was fixed at 1700 r/min.

### Gas-foaming creation of 3D NSs

The 2D PCL/PPDO NMs were expanded into 3D NSs with gas foaming technology. The pre-cut 2D NMs were immersed into NaBH_4_ (Sinopharm Chemical Reagent Co., LTD.; Beijing, China) aqueous solution at different concentrations (1 M, 2 M, 3 M, and 4 M). The hydrogen bubbles were continuously generated through the chemical reaction NaBH_4_+2H_2_O→NaBO_2_+4H_2_↑. The as-formed hydrogen bubbles penetrated the 2D NMs and expanded the 2D NMs into 3D NSs (**Video S1**). After the predetermined time point, the as-expanded 2D NMs were removed and washed 5 times to remove residual NaBH_4_. The final harvested 3D NSs were lyophilized and stored at -20 °C until further use. The gas-foaming duration and NaBH_4_ concentration were set at 20 min and 2 M, respectively, for subsequent biological experiments.

### Material characterization

(a) Morphology and structure characterization: A scanning electron microscope (SEM; TESCAN, VEGA3; Brno-Kohoutovice, Czech Republic) was used to visualize the morphology and structure of 2D NMs and 3D NSs. In preparation for SEM, the samples were sprayed with gold for 60 s to increase the electrical conductivity. An accelerated high voltage of 10 kV was adopted for image taking. ImageJ software (NIH; Bethesda, MD, USA) was used to analyse the average fiber diameter. Calculations for each specimen were based on random selection of more than 100 different sites from 3 different SEM images. The expansion height of different 3D NSs was measured with a Vernier caliper. Five independent replicates were recorded, and the average value was analysed.

(b) Fourier transform infrared (FTIR) test: A FTIR spectrometer (Nicolet 8700, Thermo Fisher Scientific; Waltham, MA, USA) was used to record the FTIR curves of different 2D NMs and 3D NSs. The scanning range and resolution were set as 500-4000 cm^-1^ and 2 cm^-1^, respectively. The absorption peaks centred at 2945 cm^-1^ and 2868 cm^-1^ were attributed to the stretching vibration of C-H. The peaks at 1724 cm^-1^ and 1472 cm^-1^ were assigned to the stretching vibration of C=O and the bending vibration of CH_2_, respectively. In addition, the three peaks at 1234 cm^-1^, 1168 cm^-1^, and 1042 cm^-1^ all belonged to the stretching vibration of C-O.

(c) X-ray diffraction (XRD) test: The XRD diffraction patterns of different 2D NMs and 3D NSs were analysed with an X-ray diffractometer (Rigaku Ultima IV, Cu Kα radiation; Tokyo, Japan). The tests were performed in the range of 5° to 60° at a speed of 5°/min.

(d) Tensile test: A universal mechanical tester (Instron 5965; Norwood, MA, USA) was used to measure the mechanical properties of different samples. The samples were clamped with a fixed gauge length of 10 mm with a preload force of 0.02 N applied. Stretching was subsequently applied at a speed of 10 mm/min until fracture occurred. Five independent replicates of each group were conducted and analysed. The load-elongation curves were recorded, and the necessary mechanical parameters, including Young's modulus, ultimate strength, and ultimate strain, were statistically calculated.

### NSC harvesting, culture, implantation, and differentiation

NSCs were isolated from Sprague-Dawley (SD) rats in wild type or green fluorescence protein (GFP)^+^ transgenic SD rats (Cyagen; Guangzhou, China). In brief, embryonic rats were sacrificed at 13-15 days gestation. The cerebral cortex of embryos was dissected and dissociated into single-cell suspensions. The cell suspension was cultured in serum-free Dulbecco's modified Eagle's medium (DMEM)/F12 (1:1, Gibco, USA) supplemented with 2% B27 (Gibco, USA), 20 ng/mL basic fibroblast growth factor (bFGF, Peprotech; East Windsor, NJ, USA), and 20 ng/mL epidermal growth factor (EGF, Peprotech). NSCs formed neurospheres in suspension and were dissociated and passaged with Accutase (Thermo Fisher Scientific) approximately once each week, with half of the medium replaced every 3 days.

After 7 days of culture, the neurospheres were centrifuged and further digested into single cells with Accutase. Single cells were seeded onto sterilized 2D tissue culture polystyrene (TCPS) plates (ibidi; Fitchburg, WI, USA) and 3D PCL/PPDO NSs, which were both precoated with 10 µg/mL poly-L-lysine (Sigma). 1×10^6^ cells/mL of NSCs were seeded onto 3D NSs from the direction perpendicular to the expanding axis. The single-cell solution was rapidly absorped into the 3D NSs due to capillary action. The seeded cells were cultured in DMEM/F12 (1:1) with 2% B27 and 10% foetal bovine serum (FBS, Thermo Fisher Scientific). After 4 h, the medium was replaced with differentiation medium consisting of DMEM/F12 (1:1) with 2% B27 and 1% FBS to identify the differentiation potential of NSCs.

### Viability and proliferation assays for NSCs

Live/dead staining was performed on NSCs by adding calcein-AM (1:1000, Beyotime; Shanghai, China) and propidium iodide (PI, 1:1000, Beyotime) to the culture medium at 37 °C for 30 min. The cells were washed three times with PBS and visualized with an ultra-high-resolution confocal fluorescence microscope (Leica DMi8; Wetzlar, Germany). ImageJ software was used to calculate the NSC survival rate.

Cell proliferation was assessed on days 1, 3, and 7 with the Cell Counting Kit-8 (CCK-8) assay (Dojindo; Mashiki, Japan), according to the manufacturer's protocol. The absorbance values of 2D and 3D groups were assessed on a microplate reader (PerkinElmer EnSight; Valenica, CA, USA) at 450 nm and normalized based on the day 1 values. Calculations were performed as described previously 21.

### Surgery and scaffold transplantation

Adult female SD rats (220-250 g) were purchased from SPF Biotechnology (Beijing, China). Rats were randomly divided into 4 groups (n = 6): Sham, SCI (no treatment after SCI), 3D NS (transplantation of 3D NSs after SCI), and 3D NS + NSC (transplantation of NSC-loaded 3D NSs after SCI). Because most human SCI are incomplete and the survival rate of animals with a large lesion gap is extremely low, the hemi-section model was applied in this study 22. Before surgery, 1×10^6^ cells/mL of NSCs were seeded on 3D NSs and cultured for 7 days in differentiation medium. Animals were anaesthetized with isoflurane. Following laminectomy, the T10 spinal cord was hemisected, and a 3 mm cord segment was removed. After haemostasis was achieved, the prepared 3D NSs were transplanted into the lesion gap, and the incision was closed. All rats received the antibiotic ceftiofur sodium (Amicogen; Jining, China) for 7 days. The bladder was manually massaged twice a day until automatic urination was restored. Cyclosporine A (Selleck; Houston, TX, USA) was intraperitoneally (*i.p.*) administered at a dose of 10 mg/kg/d until the rats were sacrificed.

### Immunofluorescence (IF) staining

Samples were fixed with 4% paraformaldehyde for 15 min, permeabilized with 0.3% Triton X-100 for 15 min at room temperature, blocked with 5% bovine serum albumin (BSA, ZSGB Bio; China) for 1 h and incubated with primary antibodies at 4 °C overnight. Fluorescence staining was performed with Alexa Fluor 488-conjugated or Alexa Fluor 594-conjugated secondary antibodies (ZSGB Bio) for 1 h, and nuclei were stained with DAPI (Beyotime) for 15 min at room temperature. Images were acquired with an ultra-high-resolution confocal fluorescence microscope (Leica DMi8) or a panoramic digital section scanning microscope (OLYMPUS VS120; Tokyo, Japan). The following primary antibodies were used: mouse anti-Nestin (1:1000, ab6142, Abcam; Cambridge, UK); mouse anti-Tuj-1 (1:1000, ab78078, Abcam); mouse anti-NeuN (1:1000, ab104224, Abcam); mouse anti-NF200 (1:600, 2836, CST; Danvers, MA, USA); rabbit anti-DCX (1:1000, ab18723, Abcam); rabbit anti-GFAP (1:1000, 12389, CST); rabbit anti-ChAT (1:200, ab181023, Abcam); rabbit anti-5-HT (1:5000, S5545, Solarbio; Beijing, China); and rabbit anti-Syn (1:200, 5297, CST).

ImageJ software was used for the semi-quantification of positive cells. Briefly, the fluorescence images were imported into ImageJ and converted into 8-bit type. The “threshold” function was used to cover positive cells, and the “measure” function was performed to collect the percentages of the covered areas. As ImageJ did not provide accurate automated cell counts for irregular shapes, manual counting was performed for the number of Tuj1^+^, GFAP^+^, and Nestin^+^ cells. To calculate the orientation of NSCs, images were processed and analysed with PAT-GEOM plugins from ImageJ 23.

### Locomotor function assessments

The open-field test and other tests were carried out to evaluate the motor functional recovery of rats following SCI (n = 6). First, Basso, Beattie, and Bresnahan (BBB) scoring was performed on 0, 1, 3, 7, 14, 21, 28, 35, 42, 49, and 56 days post-injury (dpi) according to the previous studies with modifications 22. Briefly, rats were placed in an open field (1 m × 0.8 m) for 4 min to semi-quantitatively analyse the voluntary movements of the hindlimbs on injured side by two observers blinded to the experimental groups. The scores were calculated ranging from 0 (complete paralysis) to 21 (normal locomotion).

An inclined plane test was used to evaluate the animals' grip at 4, 6, and 8 weeks post-injury (wpi). Before each measurement, the bladder of each rat was expressed. The training was performed before the formal beginning of the test. The rats were then placed on the inclined plate with a rubber pad, and the longitudinal axis of the rat was kept parallel to the longitudinal axis of the inclined plate. Each rat was assessed for 5 s five times while the height of the inclined plate was slowly raised to the maximum angle, and the average value was taken.

For the footprint analysis, animals were first trained to walk on the runway (60 cm × 10 cm), starting from a brightly illuminated box to a darkened box through a narrow channel 24. Prior to each measurement, the bladder of each rat was expressed. On the test day (4, 6, and 8 wpi), the forepaws and hindpaws of animals were stained with non-toxic red ink and blue ink, respectively. They were then placed onto the same runway covered with white paper to track the footprints. The tests were repeated if the rats turned around at any point. The rotation angle was defined as the angle of the hindpaw axis (injured side) relative to the runway axis. The interlimb coordination was represented by the relative position between the forepaws and hindpaws.

### Sensory function assessment

The adhesive removal test is a sensitive method to assess sensory deficits and recovery 24. Prior to each measurement, the bladder of the rats was expressed. At 8 wpi, each animal was put into an individual clear container without any bedding for at least 5 min. A piece of tape (15×15 mm) was then adhered to the palm of the hindpaw (injured side). The time it took for animals to sense the tape was recorded to indicate sensory function recovery after SCI.

### Magnetic resonance imaging (MRI) evaluation

MRI experiments were carried out on a 3.0 Tesla MR scanner (Siemens, MAGNETOM Verio 3.0; Munich, Germany) with a wrist coil at 4 wpi. Under anaesthesia, sagittal T2-weighted turbo spin-echo images (T2WI) of thoracic vertebra were acquired from the animals with the following parameters: repetition time (TR) = 3610 ms; echo time (TE) = 74 ms; slice thickness = 1.0 mm; field of view (FOV) = 120 mm×120 mm; average = 3.

### Electrophysiological analysis

At 8 wpi, electrophysiological examinations were performed to evaluate the functional status of sensorimotor signal conduction as previously described 2. Briefly, under anaesthesia, the sciatic nerve and sensorimotor cortex (SMC) of the animals were exposed. To record the motor evoked potentials (MEPs), the stimulating electrode was inserted into the SMC, while the recording electrode was inserted into the sciatic nerve. The stimulus voltage was 42 V, and the pulse width was 0.2 ms. Conversely, the stimulating electrode was inserted into the sciatic nerve while the recording electrode was inserted into the SMC to record somatosensory evoked potentials (SEPs). The stimulus current was 32 mA. The waveforms, amplitude, and latency of MEP and SEP were acquired and analysed.

### RNA sequencing and bioinformatics analysis

TRIzol reagent was used to extract total RNA from NSCs cultured on the 2D TCPS plate controls and 3D NSs on day 7 (n = 3). Total RNA was isolated with the RNeasy mini kit (Qiagen; Hilden, Germany), and RNA-Seq libraries were prepared with the NEBNext UltraTM RNA Library Prep Kit for Illumina (NEB; Ipswich, MA, USA). Constructed libraries were quality checked with Agilent 2200 and Qubit 3.0 (Agilent; Santa Clara, CA, USA), and sequenced on the Illumina HiSeq X ten/NovaSeq platform after passing the test (Illumina; San Diego, CA, USA). Raw data were then quality filtered to generate “clean reads” for further analysis. The differentially expressed genes (*P*-value ≤ 0.05, |Log2FC| ≥ 1) were subjected to Gene Ontology (GO) and Kyoto Encyclopedia of Genes and Genomes (KEGG) enrichment pathway analysis with Hiplot (https://hiplot.com.cn). *P*-value was adjusted through Benjamini-Hochberg false discovery rate correction for multiple testing. Differentially expressed genes (DEGs) were listed in **Table S2**.

### Statistical analysis

Statistical analysis was performed with GraphPad Prism software (version 7.0). Data are presented as the mean ± standard deviation (SD). All experiments were performed with at least 3 replicates in each group. The unpaired Student's t-test (two-tailed) was used for the mean comparison of two groups. One-way analysis of variance (ANOVA) followed by Tukey's post hoc analysis was used to compare the mean values of three groups or more. Data were analysed by two-way ANOVA for the BBB scores matched at different time points. *P* < 0.05 was determined to be statistically significant.

## Results

### Preparation and characterization of 3D NSs

**Figure 2:**
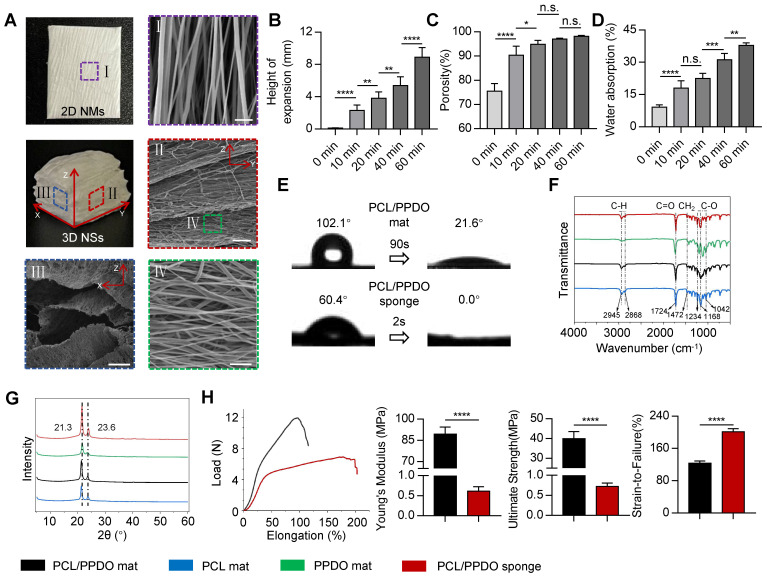
**Characterization of 2D NMs and 3D NSs. (A)** Digital photographs and SEM images of 2D NMs and 3D NSs. Scale bars, 5 μm, 100 μm, 25 μm, and 10 μm for images I-IV, respectively. Statistical analysis of **(B)** the expansion height, **(C)** porosity, and **(D)** water absorption of 3D NSs generated under different gas-forming times (n = 10). **(E)** Water contact angle of 2D PCL/PPDO NMs and 3D PCL/PPDO NSs. **(F)** FTIR spectra and **(G)** XRD patterns of 2D PCL NMs, PPDO NMs, 2D PCL/PPDO NMs, and 3D PCL/PPDO NSs. **(H)** Uniaxial mechanical testing of scaffolds (n = 5): Representative load-elongation curves; Young's modulus; Ultimate strength; and Strain-to-failure. All data are presented as the mean ± SD. ANOVA followed by Tukey's post hoc test **(B-D)**. Student's two-tailed unpaired t-test **(H)**. ^*^P < 0.05, ^**^P < 0.01, ^***^P < 0.001, and ^****^P < 0.0001 indicate significant differences. n.s. = nonsignificant.

A directional electrospinning method was first used to produce 2D PCL/PPDO NMs, which were subsequently transformed into 3D PCL/PPDO NSs through the expansion of gas bubbles generated in an aqueous solution of NaBH_4_ (**Figure 1**). A typical gas-foaming process is shown in **Video S1**. SEM images showed that the as-expanded PCL/PPDO NSs possessed a 3D laminated structure with a controllable hierarchical structure while maintaining the uniaxially aligned nanofibrous morphology (diameter 394.7 ± 99.3 nm) originating from the 2D PCL/PPDO NMs (**Figure 2A**). This result indicated that the hydrogen bubbles in the expansion process separated the nanofibers into different layers, but the necessary connections remained between adjacent layers. With increasing time during the gas-foaming process, a series of 3D NSs with different expansion heights were generated (**Figure 2B**). The initial 2D NMs displayed a dense mat-like structure with a thickness of 0.13 ± 0.01 mm, while the thickness of the 3D NSs reached 8.9 ± 1.20 mm in the NaBH_4_ (2 M) solution for 60 min (**Figure 2B** and **Figure S1**). More importantly, the porosity of 3D NSs also trended upward with increasing gas-foaming time from 75.46 ± 3.21% at 0 min to 98.68 ± 0.57% at 60 min (**Figure 2C**). However, it was found that the expanding of gas bubbles not only led to the formation of interconnected pores, but also caused the movement of nanofibers, which partly influenced their alignment and redistributed them in a 3D mode (**Figure S2**).

The hydrophilicity and wettability of engineered scaffolds play a critical role in cell-scaffold interactions in NTE. A combination of hydrophilic biomaterials and gas-foaming technology was used to improve the surface hydrophilicity of electrospun scaffolds (**Figure S3**). The gas-foaming time positively effected the water absorption capacity of scaffolds, from 9.13 ± 1.12% (2D PCL/PPDO mats) to 37.83 ± 1.21% (3D PCL/PPDO NSs), after 60 min of expansion (**Figure 2D**). Besides, after mixing PCL and PPDO into one electrospinning system, the contact angle of 2D PCL/PPDO NMs was significantly decreased from the initial 102.1° to 21.6° after 90 s (**Figure 2E**). Interestingly, a dramatically improved surface hydrophilicity was found for the 3D porous PCL/PPDO NSs generated from the gas-foaming strategy, which absorbed a droplet in less than 1 s immediately (**Figure 2E** and **Video S2**). The increased surface hydrophilicity and water absorption of 3D PCL/PPDO NSs were attributed to the significantly increased porosity after gas-foaming expansion. We also demonstrated that the NaBH_4_ concentration positively influenced the expansion height, porosity, and water absorption ability of the 3D NSs that were ultimately generated (**Figure S4**).

FTIR spectra showed that the positions of characteristic peaks had no apparent shifting after the blend electrospinning and gas-foaming process. This result indicated that no new chemical groups were generated during the creation of 2D PCL NMs, 2D PPDO NMs, 2D PCL/PPDO NMs, and 3D PCL/PPDO NSs (**Figure 2F**). XRD analysis was performed to determine the crystallinity of the above-mentioned nanofiber samples (**Figure 2G**). All four samples exhibited two sets of prominent diffraction peaks at approximately 21.3° and 23.6°, assigned to the (110) and (200) crystal planes, respectively. Importantly, the 3D PCL/PPDO NSs exhibited significantly increased diffraction peak intensity compared with other 2D NM groups, indicating that the gas-foaming technology effectively improved the crystallinity of nanofiber scaffolds.

The results from the tensile test showed that both 2D PCL/PPDO NMs and 3D PCL/PPDO NSs exhibited similar tensile load-elongation curves (**Figure 2H**). The Young's modulus for 3D PCL/PPDO NSs was significantly lower than for 2D PCL/PPDO NMs (0.61 ± 0.12 MPa *vs.* 89.34 ± 5.09 MPa). Thus, the Young's modulus of 3D NSs approached that of the native spinal cord (200-600 kPa) 2. Moreover, the 3D PCL/PPDO NSs showed notably lower breaking stress but higher breaking strain compared with the 2D PCL/PPDO NMs. Our 3D PCL/PPDO NSs also reassumed their original shape under repeated compression forces (**Video S3**). The excellent elastic recovery properties were beneficial for maintaining the stability of the porous structure during *in vivo* transplantation.

### 3D NSs enhanced the survival, neuronal differentiation, and maturation of NSCs

**Figure 3:**
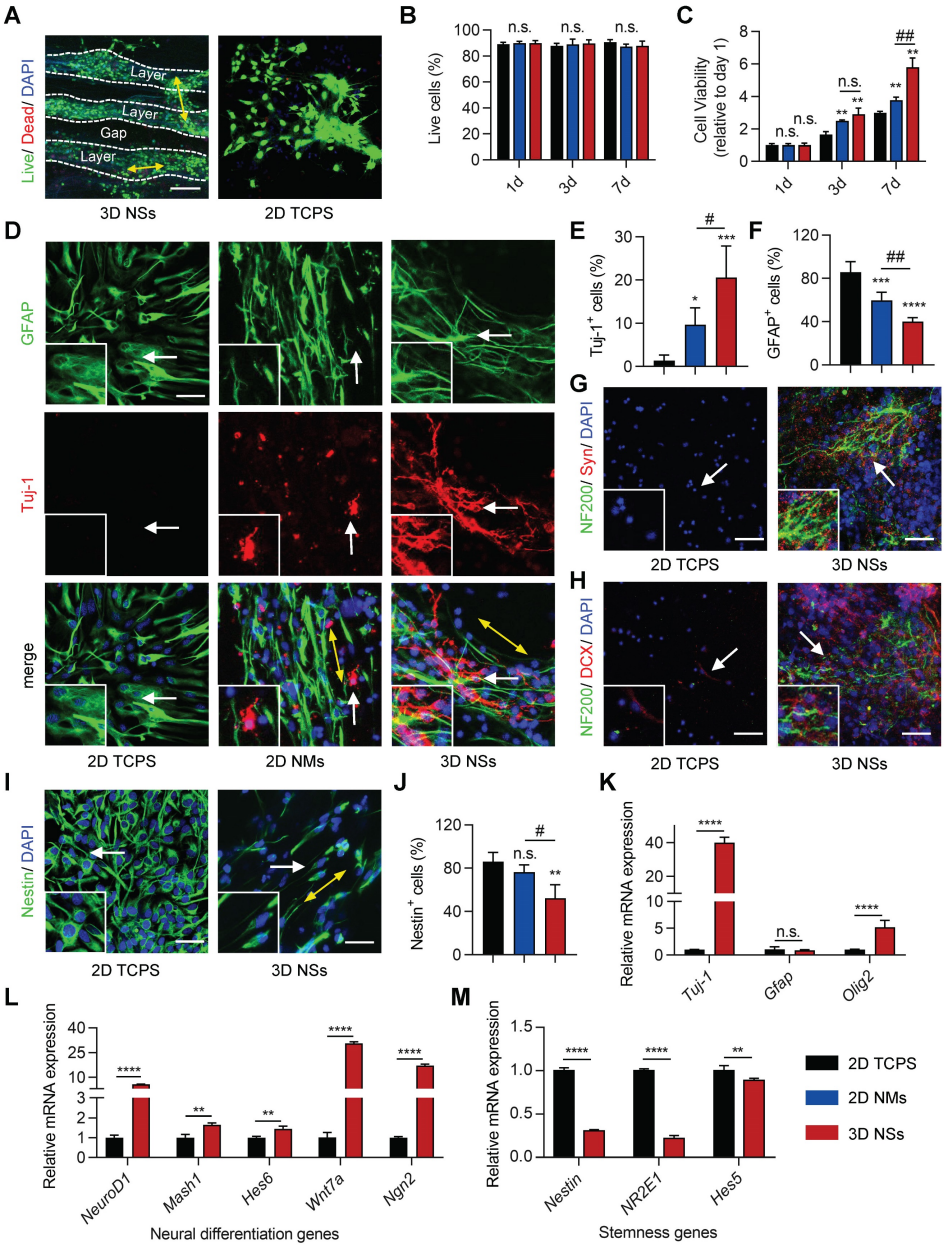
**3D NSs enhanced the survival, neuronal differentiation, and maturation of NSCs. (A)** Live (calcein-AM^+^)/ dead (PI^+^) cellular staining of NSCs cultured on 3D NSs and 2D TCPS plates on day 7 (scale bar = 75 μm). Nuclei were stained with DAPI (blue). **(B)** Quantification of the percentages of living cells on days 1, 3, and 7 (n = 4). **(C)** Cell viability of NSCs cultured on 2D TCPS, 2D NMs, and 3D NSs on days 1, 3, and 7 (n = 4) which was normalized to the absorbance (450 nm) on day 1. **(D)** Representative images of IF staining of NSCs for GFAP (green) and Tuj-1 (red) in 2D TCPS, 2D NM, and 3D NS group on day 7 (scale bar = 30 μm). The yellow bidirectional arrows indicate the direction of most cell extensions according to PAT-GEOM analysis. Enlarged views of the regions indicated with white arrows are shown in the lower-left corner to highlight the differentiated neurons. Statistical analysis of the percentages of **(E)** Tuj-1^+^ cells (n = 5) and **(F)** GFAP^+^ cells (n = 5). Representative images of IF staining of **(G)** NF200 (green)/ Syn (red)/ DAPI (blue) and **(H)** NF200 (green)/ DCX (red)/ DAPI (blue) in the 2D TCPS and 3D NS groups on day 7 (scale bar = 50 μm). Enlarged views of the regions indicated with white arrows are shown in the lower-left corner to highlight NF200^+^/ Syn^+^ and NF200^+^/ DCX^+^ cells, respectively. **(I)** Representative images of IF staining of NSCs for Nestin (green) and DAPI (blue) in 2D TCPS and 3D NS groups on day 1 (scale bar = 30 μm). Enlarged views of the regions indicated with white arrows are shown in the lower-left corner to highlight Nestin^+^ cells. **(J)** Quantification of the percentages of Nestin^+^ cells. **(K-M)** RT-qPCR to assess relative mRNA expression of **(K)** *Tuj-1, Gfap*, *Olig2*, **(L)** neural differentiation-related genes, and **(M)** stemness-related genes in the 2D TCPS and 3D NS groups (n = 3). All data are presented as the mean ± SD. Two-way ANOVA followed by Tukey's post hoc test **(B, C)**. One-way ANOVA followed by Tukey's post hoc test **(E, F)**. Student's two-tailed unpaired t-test **(J-M)**. ^*^P < 0.05, ^**^P < 0.01, ^***^P < 0.001, and ^****^P < 0.0001 indicate significant differences between the 2D TCPS group and other groups. ^#^*P* < 0.05, ^##^*P* < 0.01, and ^###^*P* < 0.001 indicate significant differences between the 2D mat and 3D NS groups. n.s. = nonsignificant.

Biocompatibility is the primary factor for an ideal transplantable biomaterial scaffold. Therefore, NSCs were seeded and cultured on 3D PCL/PPDO NSs to investigate cell-scaffold interaction. Classical and widely used 2D TCPS plates and 2D PCL/PPDO mats were used to culture NSCs as control groups. Live/dead staining showed a high survival rate (approximately 90%) of NSCs seeded on 3D NSs, 2D TCPS, and 2D NMs over the 7 days of culture (**Figure 3A-B**, **Figure S5A-B**, and **Figure S6A**). A hierarchical arrangement of NSCs was formed in the interior of 3D NSs that possessed a mean gap distance of 152.6 ± 12.5 μm (**Figure 3A** and **Figure S7**), which exhibited orientation in one layer (**Figure S5C**) and neural connections between adjacent layers. The morphology of NSCs in the 3D NSs was also examined with SEM (**Figure S5D**). In contrast, 2D NMs prevented the infiltration of NSCs to their interior (**Figure S6B**). Additionally, CCK-8 assay revealed obviously increased viability of NSCs when cultured in 3D NSs compared with those seeded on 2D plates and 2D NMs (**Figure 3C**). This result indicated that 3D NSs as “cytosponges” showed enhanced cell loading capacity.

IF staining was used to evaluate the stemness, differentiation, and maturation of the NSCs on the different scaffolds. The expression of neural-specific markers, including βIII tubulin (Tuj-1) and glial fibrillary acid protein (GFAP), was assessed to explore differentiation of NSCs on day 7. 3D NSs significantly promoted NSC differentiation towards neurons with lively outgrowing and uniaxially aligned axons along the nanofiber alignment (**Figure 3D**). Semi-quantitative analysis revealed that the fraction of Tuj-1^+^ cells (early neurons) in the 3D NSs was approximately 14 times and 2 times higher than in the 2D TCPS and 2D NM groups, respectively (**Figure 3E**), while the fraction of GFAP^+^ cells (astrocytes) in the 3D NS group was approximately 47% and 70% of the number in the 2D TCPS and 2D NM groups, repectively (**Figure 3F**). Although the NSCs growing on the surface of 2D NMs exhibited an oriented alignment and some had differentiated into neurons, no cells were observed inside the mats (**Figure S8**). Therefore, instead of simply stacking the 2D layers over each other, the hierarchical neural network formed by 3D NSs was committed to a robust cell loading capacity and pro-differenatiation properties.

We further explored whether the differentiated NSCs in 3D NSs were functional neurons. IF staining of synapsin-1 (Syn, an abundant neural protein that regulates neurotransmitter release and primarily serves as a coating protein on synaptic vesicles) and neurofilament 200 (NF200, mature neurofilaments) revealed the success construction of complex neural synaptic networks in the 3D NS group, in which more neural axons, tighter intercellular connections, and more intensive synaptic vesicles were found than those in the 2D NM group (**Figure 3G** and **Figure S9A**). IF staining of doublecortin (DCX, mainly expressed in neuroblasts and immature neurons) and NF200 also demonstrated more robust axonogenesis of NSCs in the 3D NS group, compared to the 2D NM group (**Figure 3H** and **Figure S9B**). Conversely, Nestin expression in NSCs cultured on 3D NSs was significantly lower compared with the 2D TCPS and 2D NM groups on day 1 (**Figure 3I-J** and **Figure S10**). Thus, the ECM-mimicking “cytosponges” significantly enhanced the maturation of NSCs with the ability to perform synaptic functions.

Real-time quantitative polymerase chain reaction (RT-qPCR) was also performed to assess the expression of neural-specific gene markers after 7 days in culture. The relative expression of *Tuj-1* and oligodendrocyte transcription factor 2 (*Olig2*) mRNA in the 3D NS group significantly increased by 38.9- and 4.2-fold compared with the 2D control (**Figure 3K**), respectively. This finding indicated that the ECM-mimicking topography of 3D NSs promoted the induction of the neuronal lineage of NSCs, reinforcing the IF staining results. To further explore the potential molecular mechanisms of neural differentiation, several representative neural differentiation-related genes, including neuronal differentiation 1 (*NeuroD1*), mammalian achaete-scute homologue-1 (*Mash1*), Hes family basic helix-loop-helix transcription factor 6 (*Hes6*), Wnt family member 7a (*Wnt7a*), and neurogenin 2 (*Ngn2*), were further evaluated through RT-qPCR. The mRNA levels of each of these genes significantly increased in the 3D NS group (**Figure 3L**). In contrast, some typical stemness-related genes, such as *Nestin*, nuclear receptor subfamily 2 group E member 1 (*NR2E1*), and Hes family basic helix-loop-helix transcription factor 5 (*Hes5*), were downregulated in the 3D NS group (**Figure 3M**). Therefore, the as-developed 3D NSs exhibited pro-differentiation properties, which would benefit neural reconstruction after SCI.

### Mechanism of enhanced neurogenesis from NSCs using 3D NSs

**Figure 4:**
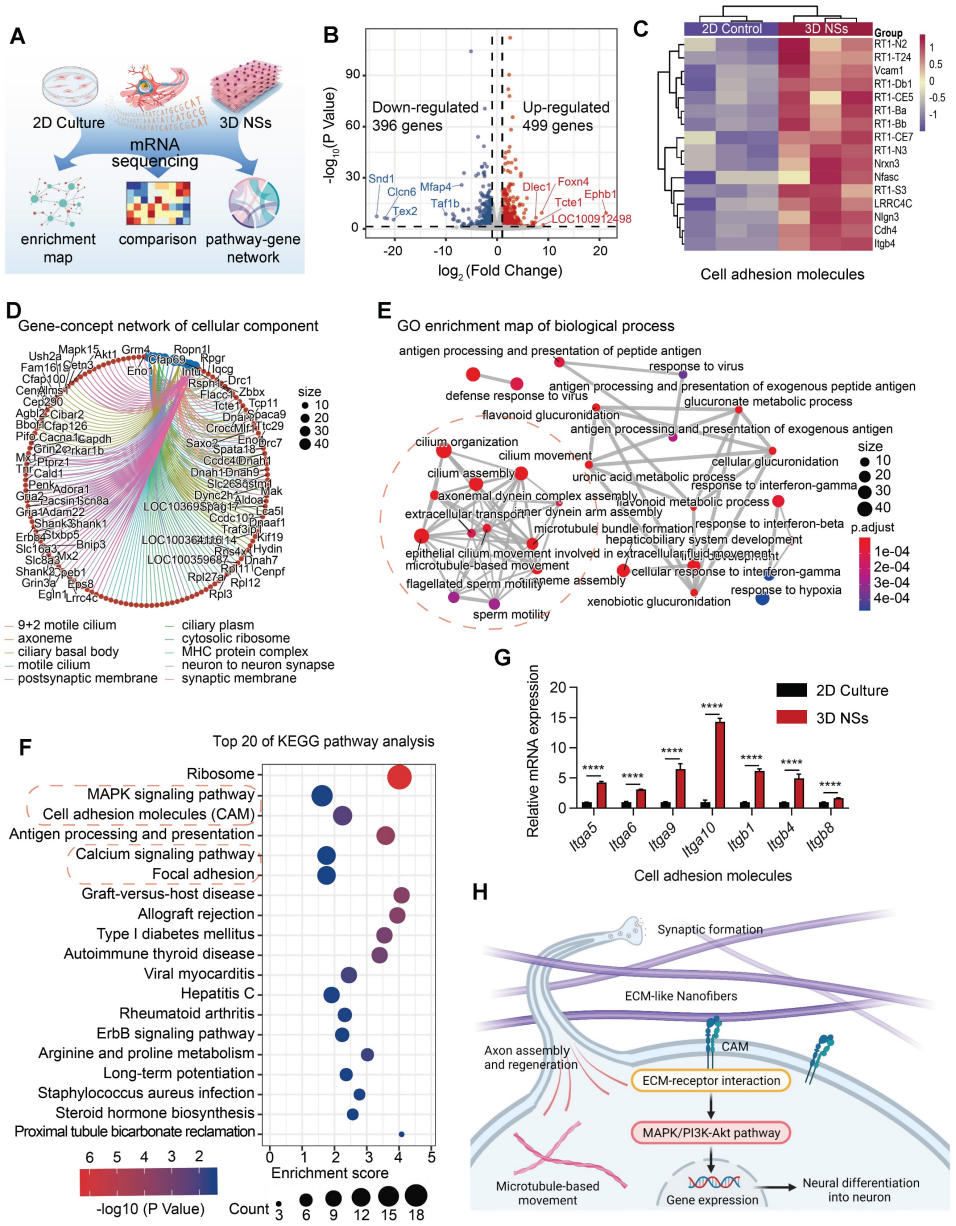
**mRNA sequencing reveals potential differentiation mechanisms of NSCs seeded and cultured on 3D NSs. (A)** Schematic illustration of the sample source for mRNA-sequence. **(B)** The volcano plot for DEGs. Red, grey, and blue points represent upregulated, nonsignificant, and downregulated genes, respectively. The symbols of the top 5 genes are indicated by red and blue lines. **(C)** Heat map showing the CAM-related DEGs between NSCs cultured on 2D control and 3D NSs. **(D)** Gene-concept network for cellular component revealed through GO analysis. The size of dots represents the number of enriched genes. Blue dots represent different enriched pathways while red dots represent different enriched gene symbols, which are connected with corresponding coloured lines. **(E)** GO enrichment map for biological process. The size of the dots represents the number of enriched genes. The adjusted *P*-value is reflected on the red and blue bars. Red dashed circles indicate the processes related to neural development and differenatiation. **(F)** Top 20 pathways from KEGG analysis. The size of dots represents the number of enriched genes. The adjusted *P*-value is reflected on the red and blue bars. Red dashed circles indicate the pathways related to neural development and differenatiation. **(G)** RT-qPCR analysis of the expression of some CAM-related genes. All data are presented as mean ± SD. The *P*-value is calculated using Student's two-tailed unpaired t-test. ^****^P < 0.0001 indicate significant differences between 2D culture and 3D NS groups.** (H)** Schematic illustration of the potential mechanisms underlying enhanced neurogenesis of NSCs cultured on 3D NSs.

The results demonstrated that the 3D NSs effectively promoted neuronal differentiation of NSCs. To examine the molecular mechanisms underlying the differences between the scaffolds, RNA sequencing was carried out to analyze DEGs of NSCs seeded on the 3D NSs and 2D TCPS plates after 7 days of culture (**Figure 4A**). The upregulated and downregulated genes (n = 499 and 396, respectively) are displayed in the volcano plot (**Figure 4B**). Many of the upregulated genes are involved in neural development of the nervous system, while many of the downregulated genes are associated with cell proliferation. For instance, the most significantly upregulated gene, ephrin receptor B1 (*Ephb1*) and its ligands, the ephrins, mediate numerous developmental processes in the nervous system 25. During neural development and injury repair, the spatially and temporally regulated expression of *Ephb1* navigates the outgrowth of axons and specifies their termination zones and synaptic partners 26. Among the downregulated genes, staphylococcal nuclease and tudor domain containing 1 (*Snd1*) plays an important role in cell proliferation and cell cycle transition by enhancing the RNA-induced silencing complex function 27. *Snd1* has also been associated with the malignant behavior of diverse types of cancer 28.

GO analysis was focused on biological process (BP), cellular component (CC), and molecular function (MF) (**Figure S11D**). The gene-concept network and enrichment map of BP revealed that the top 10 pathways targeted axoneme assembly, microtubule-based movement, and cilium organization (**Figure 4E** and **Figure S11B**). The CC analysis revealed an association with the focused network formed in the axoneme and synapse (**Figure 4D** and **Figure S11A**), which was consistent with the BP results. In addition, MF analysis indicated that NSCs on the 3D NSs were involved in cell-cell adhesion mediator activity, cell adhesion molecule (CAM) binding, and ATP-dependent microtubule motor activity (**Figure S11C**). Furthermore, KEGG enrichment revealed that the mitogen-activated protein kinase (MAPK) signaling pathway, CAM, calcium signaling pathway, focal adhesion, and other neuro-enriched pathways were involved in the interaction between 3D NSs and NSCs (**Figure 4F**). As the CAM pathway plays an indispensable role in 3D NS-induced NSC development, the related genes were selected for further analysis. RT-qPCR demonstrated that the CAM pathway was activated and the associated genes were significantly upregulated in the 3D NS group (**Figure 4G**). These results were consistent with the RNA sequencing data (**Figure 4C**).

Based on the above analysis, we propose a potential mechanism of the regulation of NSC neurogenesis by 3D NSs in **Figure 4H**. NSCs adhere to the ECM-mimicking nanofibers, resulting in the activation of CAM binding. The interaction between the CAM of NSCs and nanofibers triggers a series of downstream signaling pathways, including the MAPK pathways, which promote neuronal differentiation, axon regeneration, synaptic formation, and other critical neurophysiological processes.

### Transplantation of NSC-loaded 3D NSs enhanced neurological functional recovery in rat SCI models

**Figure 5:**
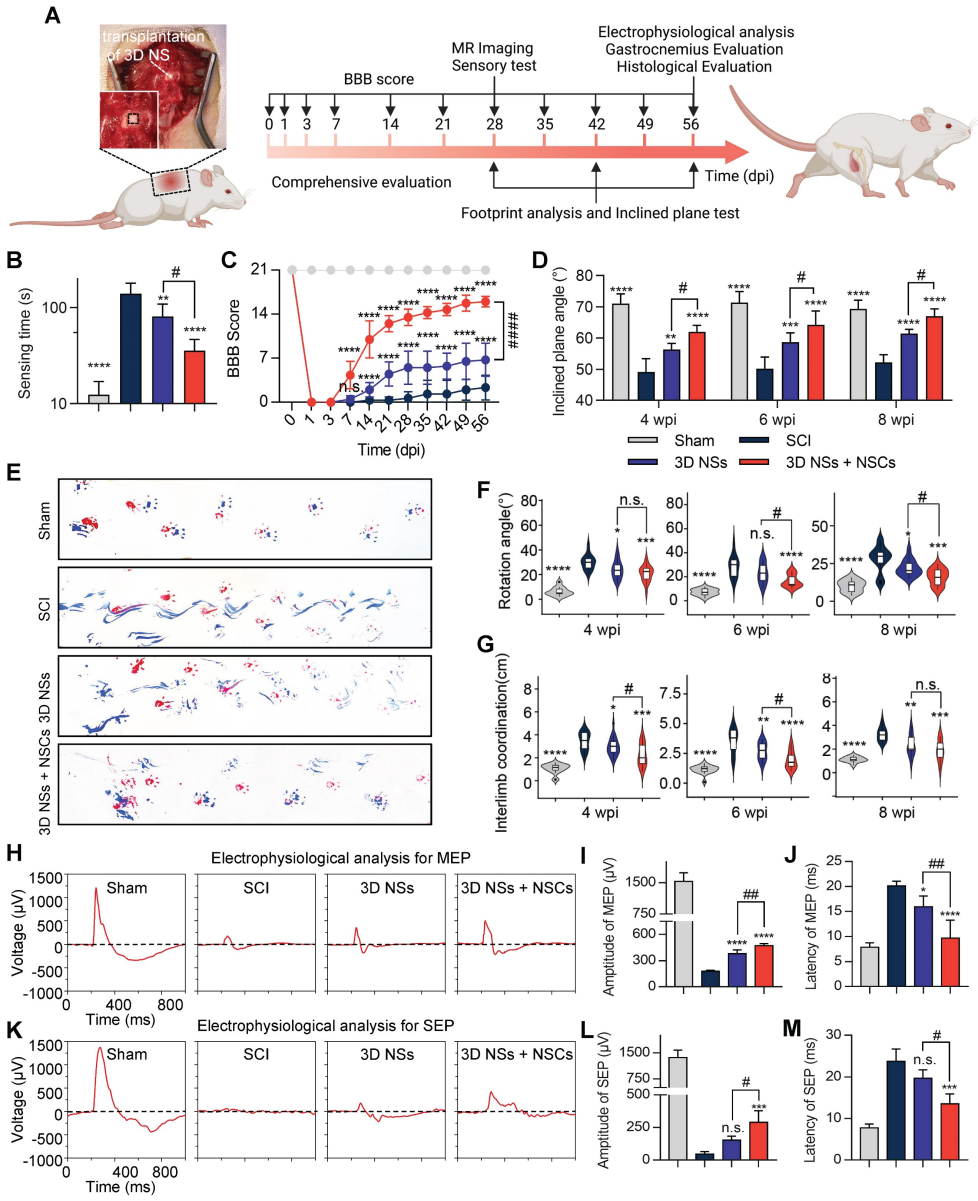
**Transplantation of NSC-seeded 3D NSs promoted neurological functional recovery in SCI rats. (A)** Schematic illustration of the animal experiment and timeline. **(B)** Sensing time for the adhesive removal test at 8 wpi for the Sham, SCI, 3D NSs, and 3D NS + NSC groups (n = 5). **(C)** BBB score throughout 56 days of treatment to evaluate locomotor functional recovery (n = 6). **(D)** Inclined plane test in the Sham, SCI, 3D NS, and 3D NS + NSC groups (n = 5) at 4, 6, and 8 wpi. **(E)** Representative footprints for the forelimbs (red) and hindlimbs (blue). Semi-quantitative analysis of **(F)** the rotation angle and **(G)** the interlimb coordination (the distance between the ipsilateral fore- and hindpaws) at 4, 6, and 8 wpi. Electrophysiological signals of **(H)** MEP and **(K)** SEP at 8 wpi. Quantification of the amplitude and latency of **(I, J)** MEP (n = 4) and **(L, M)** SEP (n = 3). All data are presented as the mean ± SD. One-way ANOVA followed by Tukey's post hoc test **(B, F, G, I, J, L, M)**. Two-way ANOVA followed by Tukey's post hoc test **(C, D)**. ^*^P < 0.05, ^**^P < 0.01, ^***^P < 0.001, and ^****^P < 0.0001 when comparing the SCI and other groups. ^#^*P* < 0.05, ^##^*P* < 0.01, and ^####^*P* < 0.0001 when comparing the 3D NSs and 3D NS + NSC groups. n.s. = nonsignificant.

To examine the *in vivo* performance of 3D NSs, the scaffolds were implanted in SD rats undergoing T10 spinal cord hemi-section (**Figure S12**). The timeline and description of the methods are shown in **Figure 5A**. Based on the practical considerations of filling the large lesion gap of spinal cords with thin 2D mats, we divided the animals into the following 4 groups: Sham, SCI (no treatment after SCI), 3D NS (transplantation of 3D NSs after SCI), and 3D NS + NSC. The walking patterns of SCI rats revealed that the combination of 3D NSs and NSCs significantly improved locomotor function and coordination (recorded in **Video S4**). The sensory testing results revealed that the animals in the 3D NS + NSC group exhibited a faster response (35.6 ± 11.3 s) compared with the others (**Figure 5B**). The BBB locomotor scores were determined during the 8 wpi. The 3D NS + NSC and 3D NS groups showed enhanced recovery with locomotor coordination regained from 1 wpi and thereafter (16.0 ± 0.8 score for the 3D NS + NSC group and 6.8 ± 2.6 score for the 3D NS group), while the SCI rats exhibited minor recovery of locomotor function (2.3 ± 2.0 score) (**Figure 5C**). Similarly, the incline plane tests revealed that the 3D NS + NSC group (61.9 ± 2.1°, 64.2 ± 4.5°, and 67.0 ± 2.3°) exhibited stronger hindlimb grip and better body coordination than the SCI (49.2 ± 4.2°, 50.2 ± 3.7°, and 52.1 ± 2.5°) and the 3D NS groups (56.3 ± 1.9°, 58.6 ± 3.0°, and 61.4 ± 1.4°) at 4, 6, and 8 wpi, respectively (**Figure 5D**). Footprint analysis was also used to test locomotor recovery at 8 wpi (**Figure 5E**). Following SCI, the coordination of the fore- and hindpaws was severely impaired with an increase in rotation angle (**Figure 5F**), relative interlimb position (**Figure 5G**), and the dragging of the ipsilateral hindlimbs, which significantly improved with 3D NS and NSCs transplantation.

Electrophysiological evaluation was also conducted to test the descending and ascending electrophysiological conductivity of the regenerated spinal cords at 8 wpi. The 3D NS and the 3D NS + NSC groups showed significantly enhanced electrophysiological recovery for both MEPs (**Figure 5H-J**) and SEPs (**Figure 5K-M**), with the amplitude rising and latency dropping.

### Histological evaluation of the regenerated spinal cord

Based on the above results, we found that the 3D NS not only acts as a vehicle to load exogenous NSCs, but also significantly activates the endogenous neural repair process. We believe the potential mechanisms of NSC-free NSs also promote neurological functional recovery in SCI animals as a result of the following features. First, most SCI-induced endogenous NSCs differentiated into astrocytes with glial scars and cavities gradually forming, which significantly obstruct axon regeneration. Here, the ECM-mimicking architectures induced the tendency of NSCs to differentiate into neurons rather than astrocytes, thus enabling cavities to be filled in and the glial scar reduced. Second, after scar reduction, the uniaxially aligned nano-structures with optimal micro-channels (~150 μm) provided important topographical cues, which functioned as “cytosponges”, to promote neuronal migration and directional extension of axons parallel to the spinal cords. Previous studies demonstrated that ~150-200 μm diameter micro-channel scaffolds were positively effective on linearly guiding axons and nerve regeneration 29, 30. Third, 3D NSs also displayed capillary action, as if for a natural sponge, contributing to the rapid absorption of nutrient solution. Fourth, the 3D NSs were composed of multiple nanofibrous layers, which had the potential acting as “biomimetic shields” to prevent epidural fibrosis and reduce the invasion of scar cells from surrounding connective tissues in the direction perpendicular to the spinal cords 31, 32. We next performed histological evaluation of transplanted spinal cords to validate our hypothesis.

**Figure 6:**
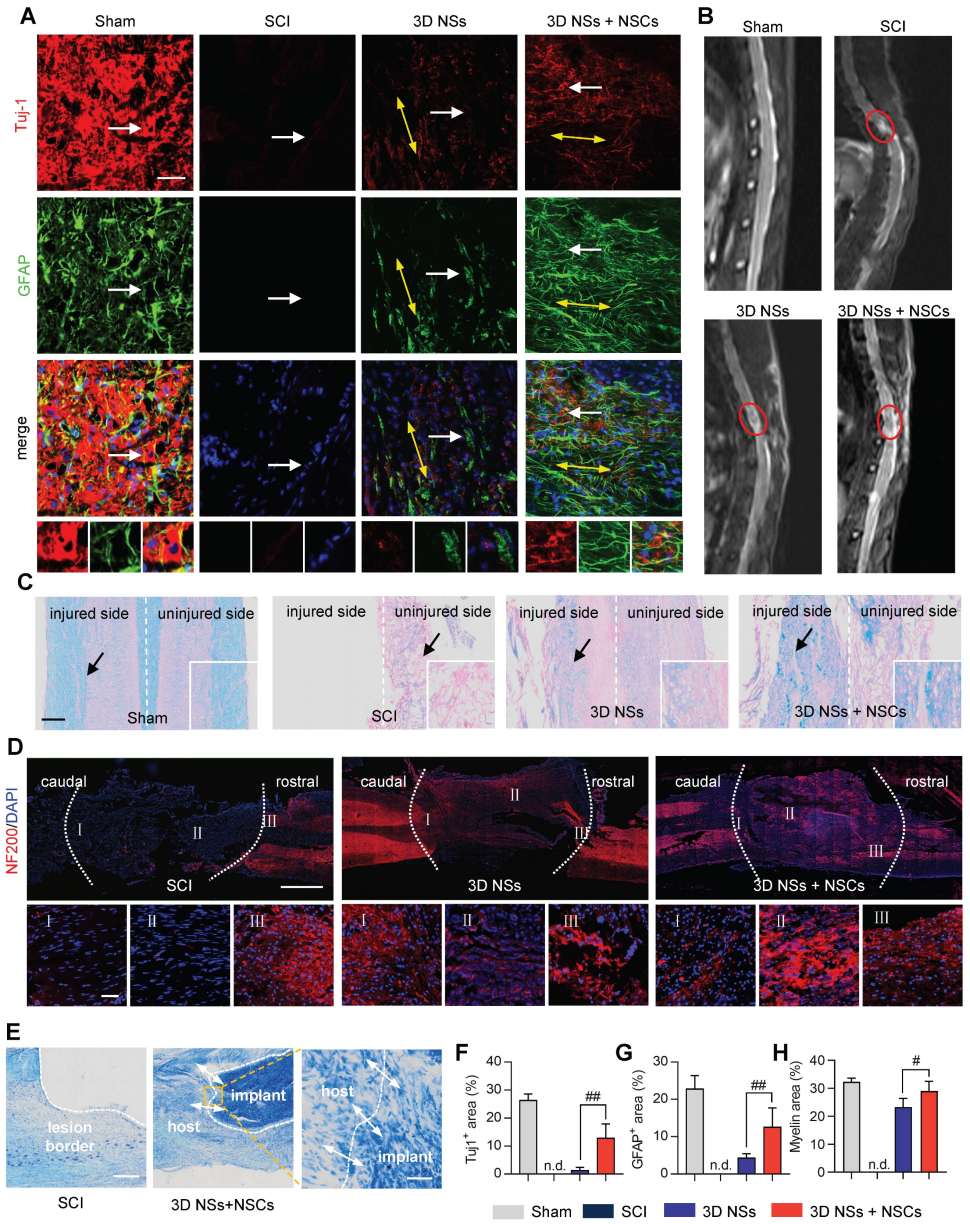
**Histological evaluation of spinal cord tissues regenerated with NSC-seeded 3D NSs during the chronic SCI stage. (A)** Representative IF images of the injured spinal cords stained with Tuj-1 (red)/ GFAP (green)/ DAPI (blue) in the Sham, SCI, 3D NS, and 3D NS + NSC groups (scale bar = 50 μm) at 8 wpi. The yellow bidirectional arrows represent the oriented neurofilaments. Enlarged views of the regions indicated with white arrows are shown in the bottom panels. **(B)** Typical MR imaging data from spinal cords in T2WI at 4 wpi. Red circles represent the injured region. **(C)** Representative LFB staining images in the Sham, SCI, 3D NS, and 3D NS + NSC groups (scale bar = 300 μm). **(D)** Representative IF staining images of the injured spinal cords stained with NF200 (red)/ DAPI (blue) in the SCI, 3D NS, 3D NS + NSC groups at 8 wpi (scale bar = 1 mm). Enlarged views of caudal, epicentral, and rostral regions are indicated as Ⅰ, Ⅱ, and Ⅲ, respectively (scale bar = 50 μm). Injured regions are highlighted with the white dotted lines. **(E)** Representative Nissl staining images in the SCI and the 3D NS + NSC groups at 8 wpi (scale bar = 500 μm). An enlarged view of the region within the yellow box is shown in the right panel (scale bar = 50 μm). The white dashed line and white bidirectional arrows represent the lesion border and cell infiltration, respectively. Quantification of the area fraction of **(F)** Tuj-1^+^ (n = 5), **(G)** GFAP^+^ (n = 5), and **(H)** myelin sheath (n = 4) at the lesion epicentre. All data are presented as the mean ± SD. One-way ANOVA followed by Tukey's post hoc test **(F-H)**. ^#^*P* < 0.05 and ^##^*P* < 0.01 when comparing the 3D NS and 3D NS + NSC groups. n.s. = nonsignificant.

NSCs were stained for Tuj-1 and GFAP at 8 wpi to assess the ability of 3D NSs to direct neuronal differentiation and maturation of exogenous and endogenous NSCs (**Figure 6A**). The largest population of Tuj-1^+^ and GFAP^+^ cells was in the 3D NS + NSC group relative to the SCI and 3D NS groups, suggesting that the aligned ECM-mimicking hierarchical structure provided specific guidance cues to enhance the differentiation of neural cells (**Figure 6F** and **6G**). Furthermore, the as-differentiated neurons established close cell contacts with the release of neurotransmitters. A large population of choline acetyltransferase (ChAT)^+^ (cholinergic neuronal marker) and 5-hydroxytryptamine (5-HT)^+^ (serotonergic neuron marker) cells were observed at the lesion site in the 3D NS + NSC group, which achieved 39% and 48% of these cell types in the Sham group, respectively (**Figure S13**).

MRI with T2WI was performed to assess the anatomical integrity of injured neural stumps (**Figure 6B**). A noticeable gap was present in the SCI group, indicating that no continuity of the spinal cord was restored without treatment after SCI. In comparison, the implanted 3D NSs enhanced the neural interaction between hosts and grafts, resulting in enhanced restoration of the anatomical structure of the transected spinal cord. These findings were also consistent with the gross view of the spinal cord, indicating that only a few connective tissues were present in the lesion area of SCI rats, while the spinal cord treated with 3D NSs showed relatively intact gross anatomy (**Figure S14A**).

Various histological staining methods were then performed to evaluate the spinal cord integrity at 8 wpi. Luxol fast blue (LFB) and hematoxylin and eosin (H&E) staining showed that the 3D NS + NSC group exhibited the highest myelin regeneration capacity and tissue preservation (**Figure 6C**, **6H**, and **Figure S14B**), which can aid in the support of metabolism and integrity in axons 33. Axonal regeneration across the lesion area did occur in 3D NS and 3D NS + NSC groups (IF staining of NF200 in **Figure 6D**). Some axons were observed extending from the nerve stump to the injury epicentre along the nanofibers. Nissl staining was then used to assess the morphology and distribution of neuronal cells. A large gap was found at the lesion site in the SCI group. The 3D sponge however provided a bridge guiding the migration of neural cells into the gap (**Figure 6E** and **Figure S14C**).

**Figure 7:**
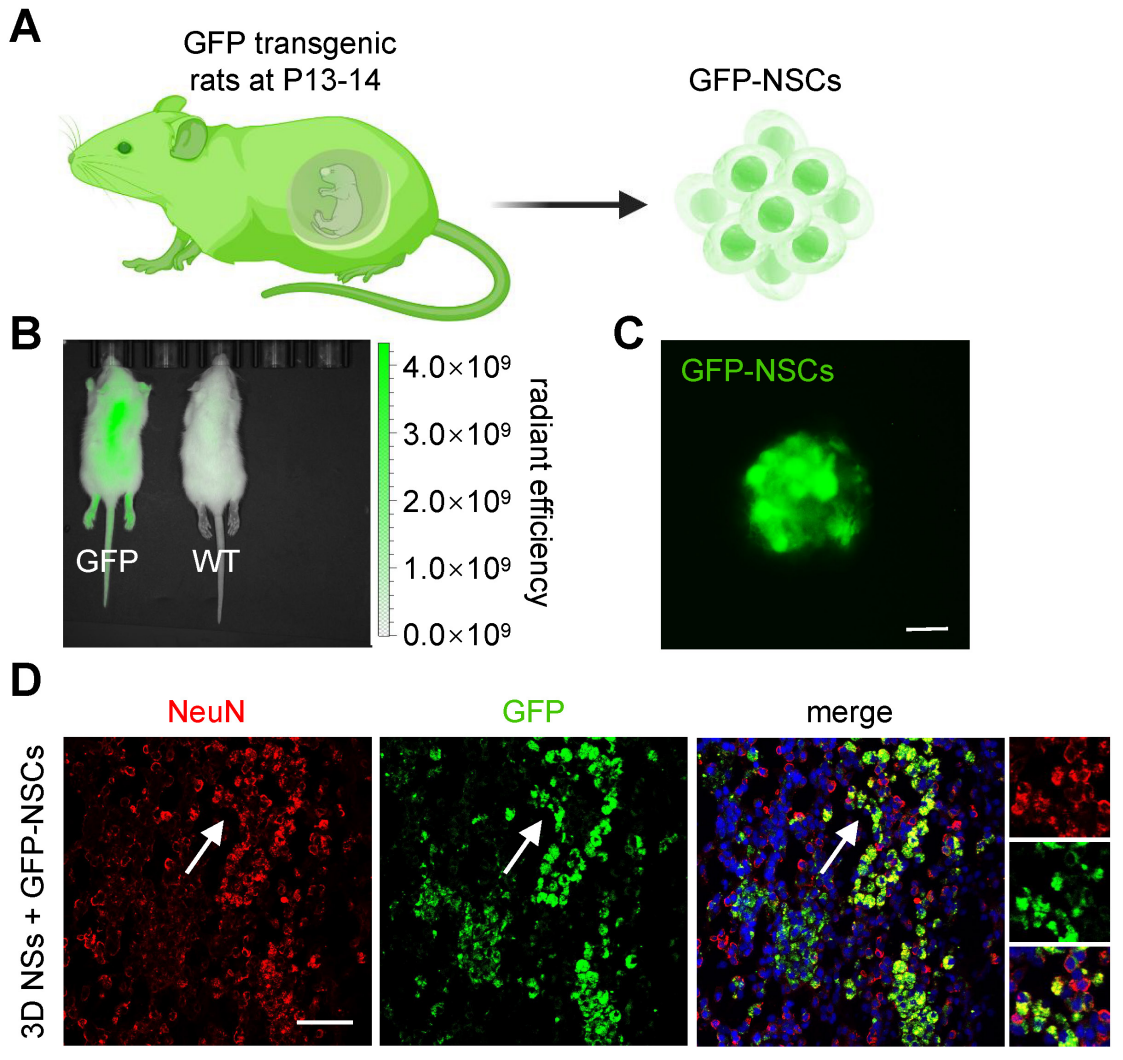
**Lineage trace of exogenous NSCs. (A)** Schematic illustration of the harvesting and culture of GFP-NSCs. **(B)** Image of bioluminescence analysis of GFP transgenic rats (left) and wild-type rats (right). **(C)** Fluorescence image of GFP-NSCs (green) (scale bar = 20 μm). **(D)** Fluorescence images of grafted GFP-NSCs in 3D NSs with NeuN staining (red), GFP (green), and DAPI (blue) (scale bar = 50 μm). The white arrow highlights NeuN^+^/GFP^+^ cells.

To trace the fate of grafted NSCs and distinguish them from host neural cells, GFP-expressing NSCs were seeded onto 3D NSs for implantation (**Figure 7A-C**). Numerous GFP^+^/ NeuN^+^ (a marker of mature neurons) cells were present in the lesion site at 8 wpi (**Figure 7D**), demonstrating that pre-differentiated exogenous NSCs were capable of survival and even maturation in the chronic phase under the shield of 3D NSs. More importantly, we found that the grafted NSCs were surrounded by host neurons, which facilitated the integration of donor NSCs with host tissues.

**Figure 8:**
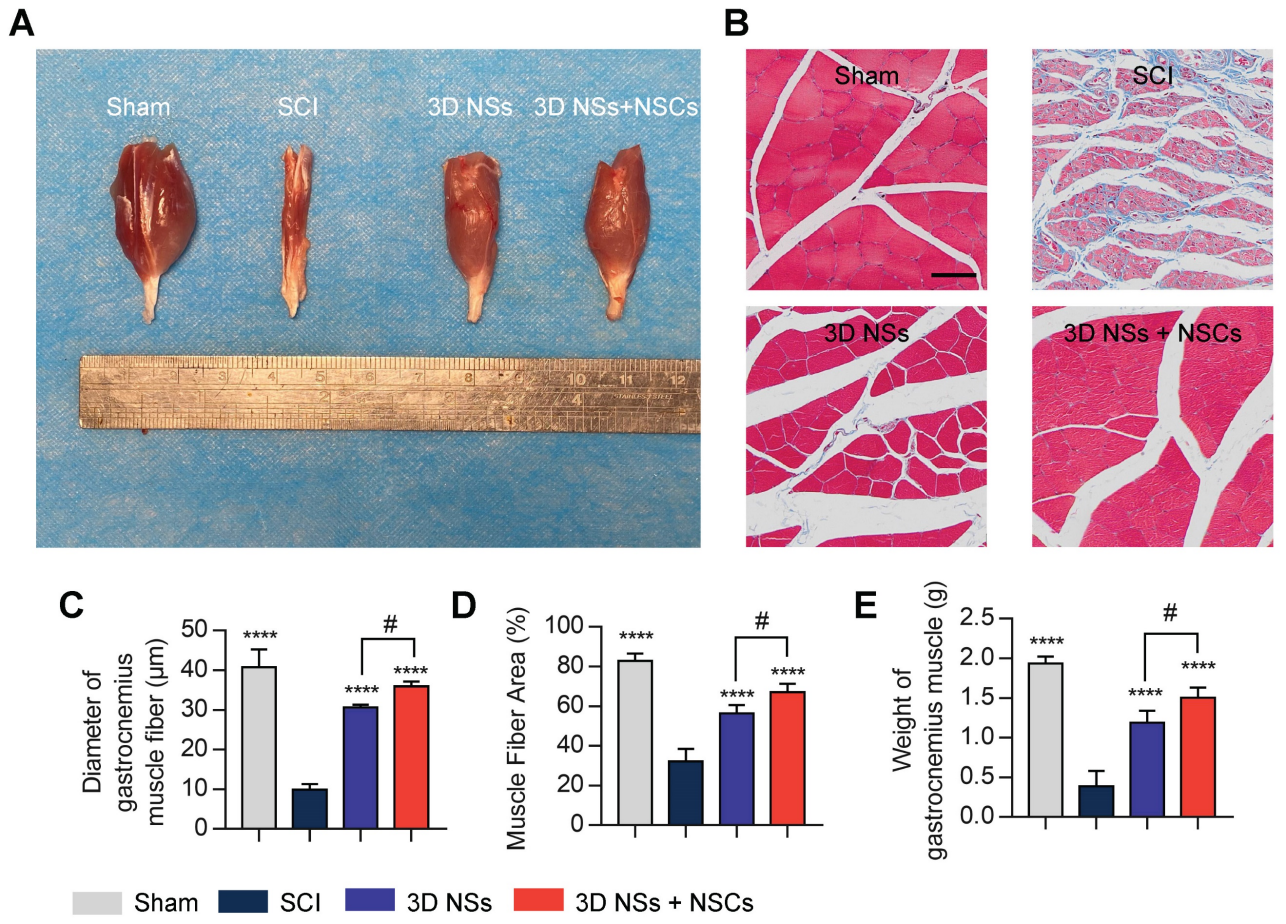
**Recovery of the gastrocnemius muscle. (A)** Representative photograph of the gastrocnemius muscle on the injured side in the Sham, SCI, 3D NS, and 3D NS + NSC groups at 8 wpi. **(B)** Representative images of Masson's trichrome staining of the transverse sections of the gastrocnemius muscles (scale bar = 50 μm). Semi-quantitative data analysis calculating **(C)** the diameter of muscle fibers, **(D)** the percentage of area, and **(E)** the weight of the gastrocnemius muscle (n = 5). ^****^P < 0.0001 indicates a significant difference between the SCI group and other groups, and ^#^*P* < 0.05 indicates a significant difference between the 3D NS and 3D NS + NSC groups. All values are presented as the mean ± SD.

Finally, gastrocnemius muscle atrophy is also a primary concern of patients with SCI, and muscle function restoration may reflect the recovery state of SCI (**Figure 8A**) 34. Masson trichrome staining indicated that the diameter and area of muscle fibers were significantly increased in the 3D NS + NSC group (**Figure 8B-D**), which was consistent with the muscle weight (**Figure 8E**). Collectively, these results further demonstrated that the biomimetic 3D NSs promoted cell survival, infiltration, differentiation, and maturation *in vivo* in the SCI rats, thus providing a histological basis for the restoration of neurological function.

## Discussion

The regeneration of neurons and axons throughout the injured site is critical for SCI repair. NTE offers a versatile and powerful platform for the construction of neural relays consisting of biomimetic nanomaterials and functional cells. Despite significant progress in developing advanced techniques to generate 3D oriented scaffolds for cellular delivery, such as electrospun scaffolds and hydrogels, some certain limitations persist, necessitating further refinement for NTE applications 6, 35, 36. Primarily, previously reported electrospun scaffolds have been collected into 2D pattern, which poses challenges on precisely controlling layer spacing for effective cell infiltration 37. Additionally, the use of nanofiber yarns produced via textile fabrication methods, comprising thousands of well-aligned electrospun nanofibers, unavoidably enhances the mechanical strength of the scaffolds to several hundred MPa, which is far stiffer than native spinal cords 38, 39. While post-processing methods like ultrasonication can aid in elevating 2D scaffolds into 3D ones, this approach may adversely impact the molecular weights and mechanical properties of nanofibers due to insufficient thickness and irregular geometry 6. Furthermore, existing fabrication techniques for anisotropic hydrogels demand further improvement. Notably, while 3D bioprinting is a valuable method in creating scaffolds with complex structures and encapsulated cells, the high cost of devices and limited printing resolution hinder precise control over the nanoscale architecture of aligned pores or channels in the scaffolds 13, 40, 41. An alternative simple approach involves the use of unidirectional freezing to fabricate 3D-oriented scaffolds 42. However, this method does not permit cell encapsulation nor provide control over construct shape and pore size. Ion diffusion has also been explored as a 3D fabrication strategy via inducing the oriented diffusion of electrolyte ions into the polymer solution, leading to continuous hydrogel formation 43, 44. Nonetheless, this approach is limited to polymers that react with ions, and it remains challenging to control pore size and topographical nanostructures. Another avenue for preparing 3D biomimetic scaffolds involves the utilization of aligned decellularized tissues (*e.g.*, native aponeurosis and spinal cords) 45, 46. However, their clinical application is severely constrained by their animal origin, undefined composition, batch-to-batch variability, poor tractability, and high cost. Thus, the pressing challenge is to devise a simple, controllable, and stable technology for the production of 3D biomimetic scaffolds tailored to neural regeneration.

In recent years, gas-foaming techniques as one of the important post-processing strategies were introduced to produce 3D electrospinning scaffolds 47. Although Jiang *et al.* reported that the 3D scaffolds contributed to cell infiltration in subcutaneous grafts, it has not been systematically optimized and developed for complex central nervous system (CNS) regeneration, which only had extremely limited regenerative capacity 14. For example, the gap distance of their scaffolds was mainly distributed in the range of 20-30 μm, while the most effective spacing for linear neural tract guidance and neural connections was approximately 200 μm 30, 48. Besides, the excellent cellular infiltration ability of scaffolds is actually a double-edged sword. How to promote neuronal migration and differentiation while reducing glial cell and inflammatory cell infiltration and providing a stable microenvironment for neural regeneration is worth further exploration. Therefore, it is of significant importance to understand the neuronal pattern of exogenous and endogenous NSCs in 3D NSs and explore the mechanism that sufficient numbers of regenerating axons cross the lesion site and remake functional synapses with neurons in the targets.

In this study, a novel 3D PCL/PPDO NSs was developed through the combination of directional electrospinning and modified gas-foaming technology, effectively overcoming the above obstacles in CNS regeneration. With this approach, the imparted anisotropic cues of biomimetic scaffolds were entirely preserved, presenting a uniaxially aligned nano-architecture and a highly controllable hierarchical structure. The ultimate goal is for 3D NSs to serve as anisotropic “cytosponges” with biomimetic porous structures, outstanding hydrophilicity, and reasonable mechanical performance. Although the gas-foaming treatment partly reduces the nanofiber orientation of 3D NSs which may have some impact on the directional axon growth, the overall effectiveness of the scaffold in promoting neural regeneration is greatly enhanced. It is essential to recognize that the spinal cord is not entirely uniformly oriented, which also contains other orientations, complexities, and networks that enable its intricate functions. Herein, 3D NSs created a generally aligned but interconnected hierarchical network to better mimic the native spinal cord. Compared with the simple and dense 2D NMs, the porous nanostructure (98.68 ± 0.57% porosity) of 3D NSs significantly enhanced the penetration of attached neurons, oxygen and nutrient exchange, and metabolite emission, which significantly promoted neural circuit reorganization and integration. Importantly, 3D NSs directedly recruited neural cells parallel to spinal cords, while preventing fibrous collagen capsules as described by others previously 14.

The “cytosponges” were demonstrated to induce alignment, facilitate migration, promote neuronal differentiation, and even phenotypic maturation of NSCs without any supplemental neurotrophins or additives. However, it was still challenging to maintain a high NSC survival rate over an extended period of time *in vivo* because of the deleterious microenvironment at the site of the lesion 2, 49. Here, the ECM-mimicking structure of the 3D NSs can provide a more suitable environment for cell survival and functionalization. Even after 8 weeks, GFP-positive NSCs (exogenous cells) were still observed in the lesion areas. Therefore, neurological function and anatomical structure in SCI rats were significantly improved under the treatment with 3D NSs. Overall, the 3D biomimetic NSs provide significant neuroprotection and guidance to the newly formed “neural relay”, promoting axon regeneration, myelinogenesis, and synaptic reconnections *in vivo*.

The potential mechanism of the neuronal response to 3D NSs was elucidated through mRNA sequencing. When NSCs attach to ECM-mimicking nanofibers, growth cones and focal adhesion are involved in the regulation of cell behaviour 13. Growth cones are the sensitive structures at the apical end of growing axons, which consist of microtubules and actin filaments. These microtubules and filaments enable the perception of morphology-associated cues by forming a complex interacting meshwork. To minimize distortion of the cell cytoskeleton caused by anisotropic cues, microtubules grow and shrink dynamically to align with the directional nanofibrous structure 50. In addition, focal adhesions, as complex protein clusters integrating the cytoskeleton with the adhesion substrates through CAM activation, have also been shown to play an essential role in mediating neurite outgrowth along the biomimetic scaffolds 51. Integrin binding, for example, is an interaction with the cell which initiates downstream signal transduction through calcium signaling activation, and causes gradual changes in cell morphology and biological function 51, 52. Therefore, we presume that growth cones and focal adhesion are the bridges between the ECM-mimicking cues of 3D NSs and neural cell behaviors by activating the CAM-based MAPK/phosphatidylinositol 3 kinase (PI3K)-protein kinase B (AKT) signaling cascade. The potential mechanism was also verified with our previous report about 3D electrospinning scaffolds* in vitro*, and the *in vivo* performance of biomimetic scaffolds was explored in this study 53.

Compared with other biomaterial scaffolds, the exceptionally high porosity of 3D NSs endows them with a higher cell load factor. Their unique structures provide a more suitable microenvironment, allowing loaded NSCs and endogenous cells to migrate and communicate freely in the direction parallel to the spinal cords while preventing cell invasion from other directions. The anisotropic ECM-mimicking architectures significantly restore the transmission of sensory and locomotor electrophysiological signals and improve the neurological locomotor function after SCI. Therefore, the universal strategy for elevating dimensions dramatically broadens the application boundaries of traditional electrospinning scaffolds, providing a new prospect in the regeneration of CNS and other anisotropic tissues.

## Conclusion

This study provided the methods of a universal strategy to transform nanofibrous scaffolds from 2D to 3D for SCI therapy. The 3D NSs exhibited uniaxially aligned ECM-mimicking architecture and controllable hierarchy, which induced the rapid differentiation of NSCs into neurons with synaptic networks mediated by CAM activation. The sponge-like porous structure was instrumental for the interaction between exogenous and endogenous NSCs, which contributed to neural relay reconstruction and electrophysiological signalling transmission. Therefore, 3D NSs, as novel “cytosponges”, represent a promising cell delivery system to facilitate neurogenesis and functional restoration in SCI.

## Supplementary Material

Supplementary methods, figures, table 1, table 2 title, video legends.

Supplementary table 2.

Supplementary video 1.

Supplementary video 2.

Supplementary video 3.

Supplementary video 4.
