# Supplementary material for: Three-dimensional nanofibrous sponges with aligned architecture and controlled hierarchy regulate neural stem cell fate for spinal cord regeneration
Source: Theranostics. 2023 Aug 28;13(14):4762–80. doi: 10.7150/thno.87288 (PMC10526661; doi:10.7150/thno.87288)
Supplement: Supplementary file 1 — Supplementary methods, figures, table 1, table 2 title, video legends. [file thnov13p4762s1.pdf]

Supplementary Materials for

Three-dimensional nanofibrous sponges with aligned architecture and controlled hierarchy regulate neural stem cell fate for spinal cord regeneration

This PDF file includes:

Supplementary Methods;

Figures S1 to S14;

Tables S1 and S2;

Videos S1 to S4.

Supplementary Methods

Water absorption test

The initial weight W_0 (g) of different 2D NMs and 3D NSs was measured in the dry state. The samples were then soaked in 20 mL distilled water for 1 h, removed and placed on a filter for 10 min. The water on the surface of the samples was allowed to drip off under the action of self-weight, and the wet weight W (g) of the corresponding samples was subsequently recorded. The water absorption capacity was calculated according to the following formula: Water absorption (%) = $(W-W_0)/W_0 \times 100\%$.

Contact angle test

The water contact angle of different 2D NMs and 3D NSs was recorded and analysed through a contact angle tester (Shanghai Xuanyichuangxi Industrial Equipment Co., Ltd., XG-CAMD3; Shanghai, China). Briefly, 2 μL deionized water was dropped on the surface of different samples, and the process of droplet absorption was recorded through both photographs and videos.

Porosity test

The porosity of the different samples was calculated based on the change in their volumes by the following formula: Porosity (%) = $(V-V_0)/V \times 100\%$. V (cm^3) refers to the whole volume of samples calculated by measuring the length, width, and height; V_0 (cm^3) is the occupied volume of PCL and PPDO polymers in the corresponding sample. V_0 was calculated from the formula: $V_0 = m_0/\rho_0$. Note: m_0 (g) refers to the total mass of the PCL and PPDO polymer materials; ρ_0 (g/cm^3) refers to the density of PCL (1.145 g/cm^3) and PPDO (1.100 g/cm^3) after mixing.

SEM analysis of NSCs

SEM was utilized to examine the morphology of NSCs on 3D NSs after 7 days in culture. Briefly, the NSCs-3D NSs complex was fixed in 2.5% (w/v) glutaraldehyde (Macklin, China) at 4 °C for 60 min. After dehydration with a series of graded ethanol, the cell-scaffold constructs were lyophilized and coated with gold for SEM.

RT–qPCR analysis

Total RNA was extracted from NSCs seeded on control 3D NSs and 2D TCPS plates using TRIzol reagent (Thermo Fisher Scientific; Waltham, MA, USA) on day 7. The harvested RNA was reverse-transcribed into complementary DNA (cDNA) using a ReverTra Ace qPCR RT Kit (TOYOBO; Osaka, Japan), and RT–qPCR was performed with TB Green Premix Ex Taq (Takara; Kusatsu, Japan) on a 480II Real-Time PCR Detection System (Roche; Basel Switzerland). The reaction protocol was performed as follows: 95 °C for 30 s, 40 repeated cycles of 95 °C for 5 s, and 60 °C for 31 s. Three independent replicates were conducted for each sample. GAPDH was used to normalize the mRNA expression. The results are presented using a comparative $2^{-\Delta\Delta Ct}$ method. All the primer sequences are presented in Table S1.

Perfusion and tissue preparation

Under deep anaesthesia with 2.5% Avertin (300 mg/kg, i.p.), all animals were intracardially perfused with phosphate buffer solution (PBS) followed by a 4% paraformaldehyde solution at 8 wpi. The scaffold-spinal cord samples were stripped from vertebrae and postfixed overnight in 4% paraformaldehyde, and the harvested samples were dehydrated in 30% sucrose at 4 °C. Longitudinal and transverse sections were sliced (10 μm thickness) at the lesion epicentre with a cryostat (Leica, CM 1900; Wetzlar, Germany).

Histological evaluation.

For LFB staining of the myelin sheath, longitudinal cryosections were incubated in 0.1% LFB staining solution (Servicebio; Wuhan, China) at 65°C for 4 h. The slides were then differentiated and counterstained with 0.05% lithium carbonate and cresyl violet solution. Optical microscopic observation showed that the nerve myelin sheath was blue with a light red background, which was quantitatively analysed with ImageJ software.

For Nissl staining of neurons, the sections were treated with Nissl staining solution (Servicebio) for 5 min and then mounted with neutral balsam. Under the microscope,

dark blue granular Nissl bodies were seen surrounding the nuclei of neurons with a light blue background.

H&E staining was performed for the general assessment of histological morphology. Briefly, dewaxed sections were stained with haematoxylin solution for 3-5 min, followed by haematoxylin differentiation solution; they were then blued with Scott's tap water substitute and rinsed with tap water. After treatment with 85% and 95% ethanol for 5 min, the samples were stained with eosin dye for 5 min. The blue nucleus and red cytoplasm were visible under bright field microscopy.

Supplementary Figures

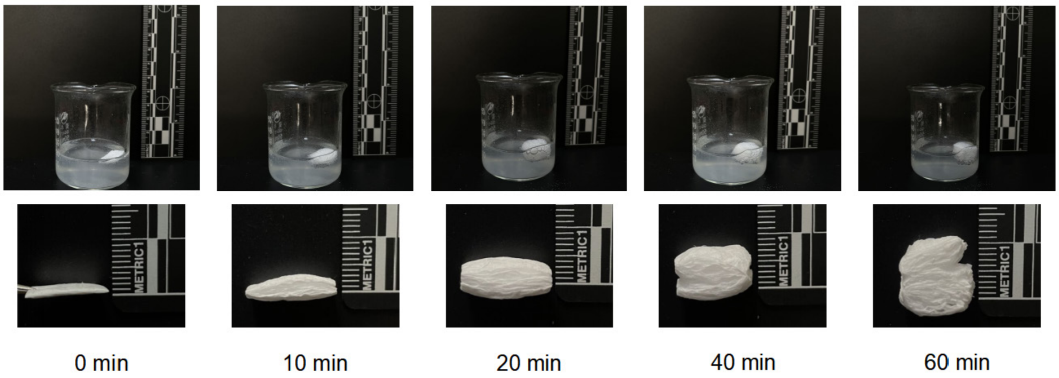

Figure S1. Photographs of sponge at 0 min, 10 min, 20 min, 40 min, and 60 min expansion times.

Figure S2. Nanofiber orientation (°) in the **(A)** 2D NM and **(B)** 3D NS group.

Figure S3. The water contact angles of the PCL mat, the PPDO mat, and the PCL/PPDO mat.

Figure S4. Characteristics of the PCL/PPDO sponge with different $NaBH_4$ concentrations. **(A)** Height of expansion, **(B)** porosity, **(C)** water absorption, and **(D)** photographs of the sponge with $NaBH_4$ concentrations of 0 M, 1 M, 2 M, 3 M, and 4 M. All values are presented as the mean ± SD. One-way ANOVA followed by Tukey's post hoc test (**A-C**). $^{**}P < 0.01$, $^{***}P < 0.001$, and $^{****}P < 0.0001$ when compared with each other.

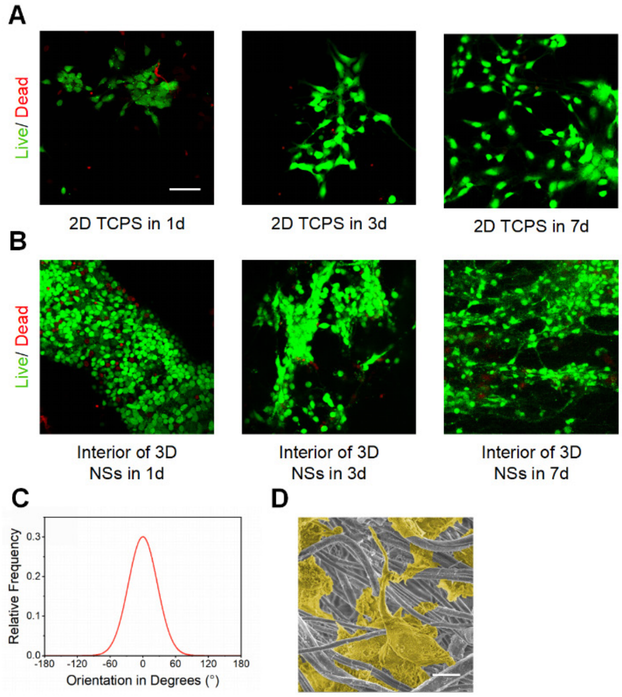

Figure S5. Live/dead staining of NSCs on 2D TCPS and 3D NSs. Live (calcein-AM$^+$)/ dead (PI$^+$) staining of NSCs seeded on **(A)** 2D TCPS and **(B)** 3D NSs for 1 day, 3 days, and 7 days. Scale bar: 30 μm. **(C)** Orientation of NSCs cultured on 3D NSs based on PAT-GEOM analysis. **(D)** Representative SEM images of NSCs seeded and cultured on 3D NSs on day 7. The cells are highlighted with yellow pseudo-colour (scale bar = 3 μm).

Figure S6. Live/dead staining of NSCs on 2D NMs. Representative images from **(A)** the surface and **(B)** interior of 2D NMs cultured for 1 day, 3 days, and 7 days. Scale bar: 50 μm.

Figure S7. Gap distance of micro-channels in 3D NSs.

Figure S8. (A) Representative fluorescence images of NSCs in the interior of 2D NMs cultured for 7 days, and stained for Tuj-1 (red)/GFAP (green)/DAPI (blue). Scale bar: 50 μm. **(B)** Comparison between the surface and interior of 2D NMs. Scale bar: 100 μm.

Figure S9. Representative images of IF staining of NSCs for **(A)** NF200 (green)/ DCX (red)/ DAPI (blue) and **(B)** NF200 (green)/ Syn (red)/ DAPI (blue) in the 2D NM group on day 7 (scale bar = 50 μm). Enlarged views of the regions indicated with white arrows are shown in the lower-left corner to highlight the neurons $NF200^{+}/DCX^{+}$ and $NF200^{+}/Syn^{+}$, respectively.

Figure S10. Representative images of IF staining of NSCs for Nestin (green)/ DAPI (blue) in the 2D NM group on day 1 (scale bar = 30 μm). Enlarged views of the regions indicated with white arrows are shown in the lower-left corner to highlight $Nestin^{+}$ cells.

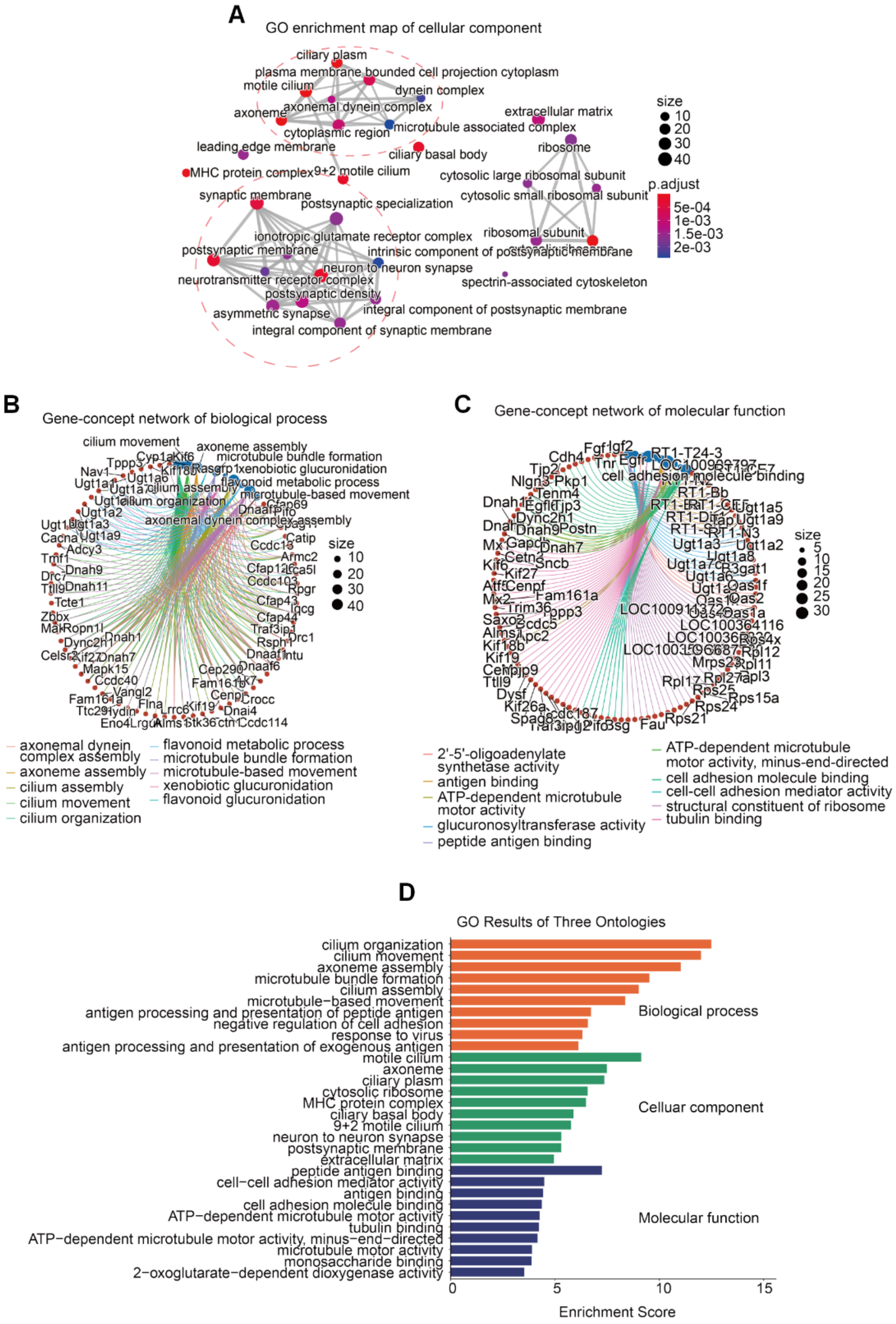

Figure S11. GO results of NSC differentiation based on mRNA sequencing. (A) GO enrichment map for cellular component. The adjusted *P*-value is reflected in the red and blue bars. Gene-concept network derived from GO analysis for **(B)** biological process and **(C)** molecular function. The size of dots represents the number of enriched genes. Blue dots represent different enriched pathways while red dots represent different enriched gene names, which are connected with corresponding coloured lines. **(D)** Overview of three ontologies from GO results.

Figure S12. Surgical methods for 3D NS transplantation. (A) The normal and intact spinal cord of rats at the T10 level. **(B)** The hemisected gap at a length of 3 mm, which is highlighted with the black dashed line. **(C)** Transplantation of the 3D NS into the injured gap highlighted with the black dashed line.

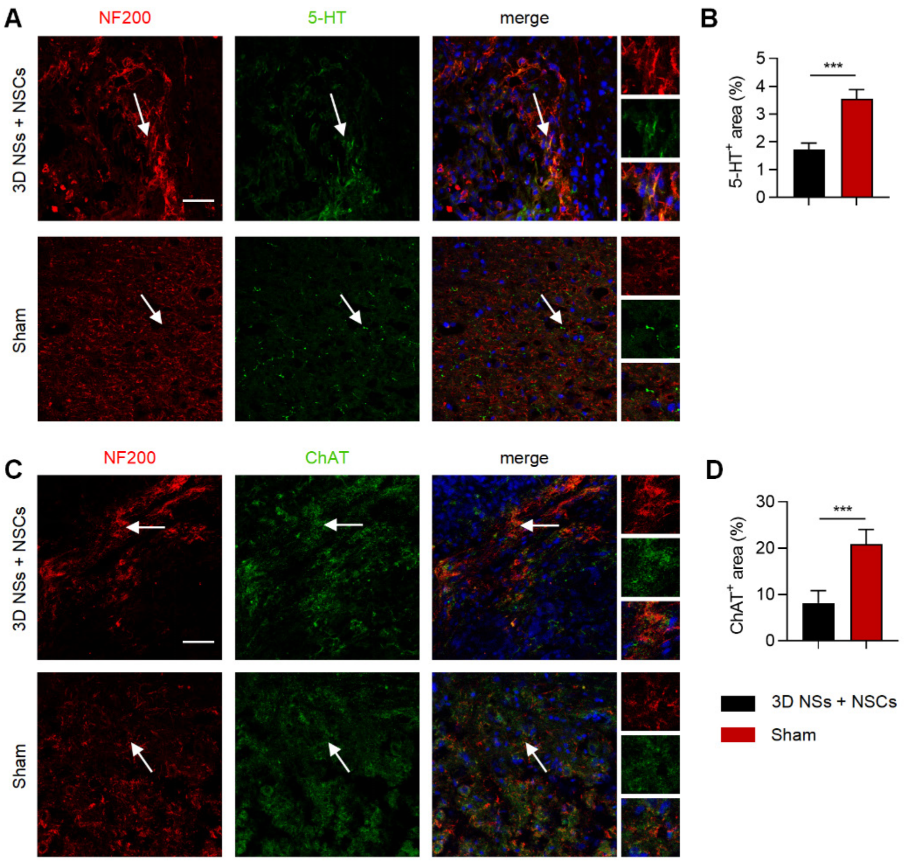

Figure S13. Representative fluorescence images of the spinal cord sections stained with **(A)** NF200 (red)/ 5-HT (green)/ DAPI (blue) and **(C)** NF200 (red)/ ChAT (green) / DAPI (blue) in the 3D NS + NSC and Sham groups (scale bar = 50 μm). Enlarged views of the regions marked with white arrows are shown in the right panel. Quantification of the area fraction of **(B)** the 5-HT^{+} (n = 4) and **(D)** ChAT^{+} (n = 4) in the lesion area.

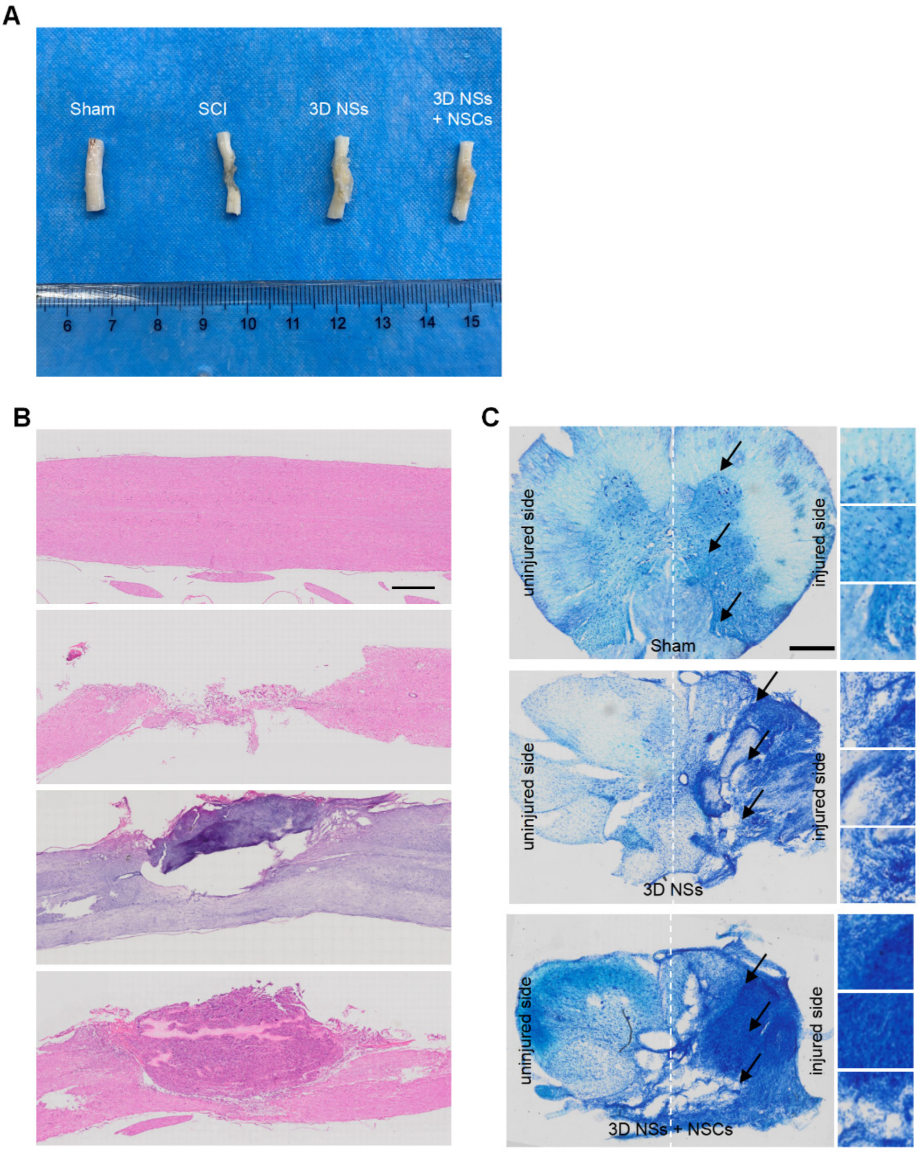

Figure S14. Histological evaluation of the spinal cord in the chronic phase of SCI. (A) 3D NS–treated spinal cords photographed at 8 wpi. **(B)** Representative H&E staining images of longitudinal spinal cord sections in the Sham, SCI, 3D NS, and 3D NS + NSC groups at 8 wpi. **(C)** Representative Nissl staining images of transverse spinal cord sections in the Sham, 3D NS, and 3D NS + NSC groups at 8 wpi. Scale bar: 300 μm.

Rattus Gene	Primer	Sequence (5'-3')
Tuj-1	Forward	CAACTATGTGGGGGACTCGG
	Reverse	TGGCTCTGGGCACATACTTG
Olig2	Forward	GTCGCCAGAACCCGATGAT
	Reverse	ACACGGTTCCTCCTGTGAAG
Gfap	Forward	TTGACCTGCGACCTTGAGTC
	Reverse	GAGTGCCTCCTGGTAACTCG
NeuroD1	Forward	AGCCCCCTAACTGATTGCAC
	Reverse	TCGGTGGATGGTTCGTGTTT
Mash1	Forward	CCACCATCTCCCCCAACTAC
	Reverse	GGAGGAGTAGGACGAAACCG
Hes6	Forward	GAGTCCCCATTGTCCAGTCC
	Reverse	CACCCGGTTTAGTTCAGCCT
Wnt7a	Forward	GGGTGCGAGCATCATCTGTA
	Reverse	AAACTGACACTCGTCCAGGC
Ngn2	Forward	CCAACTCCACGTCCCCATAC
	Reverse	TGCCAGTAGTCCACGTCTGA
Nestin	Forward	CTTCTCTTGGCTTTCTGGACCC
	Reverse	CAAGGGTATTAGGCAAGGGGG
NR2E1	Forward	CTCTACTTCCGTGGGCACAA
	Reverse	TTCATGGGGATACTTGGGCG
Hes5	Forward	AGGACTACAGCGAGGGTTACT
	Reverse	CCGCTGGAAGTGGTAAAGCA
Itga5	Forward	GAATACCAGCCGTTCAGCCT
	Reverse	GGCAGGATTTGGTAGGGCAT
Itga6	Forward	ACAACGCCTTTCTTCGGCTA
	Reverse	GGATTTCTGGCGGAGGTCAA
Itga9	Forward	GGGCGGTGAAGAACATCTCT
	Reverse	TGAAGGACACATTGGCGTCG
Itga10	Forward	AGCATCACCCACGCCTATTC
	Reverse	CCAGGTGGCCTTTGGTACAT
Itgb1	Forward	TTGCCTTGCTGCTGATTTGG
	Reverse	AGTTGTCACGGCACTCTTGT
Itgb4	Forward	AGAGTCCCAGCCATACCGAT
	Reverse	ATAGCCTCTCTCTCGGGTCC
Itgb8	Forward	GCGTTTGTGGCAGGAATTGT
	Reverse	CAGCACAGGAGGCTTTACAC
Gapdh	Forward	TCTCTGCTCCTCCCTGTTCT
	Reverse	ATCCGTTCACACCGACCTTC

Table S1. RT-qPCR primers

Table S2. Differentially expressed gene list of mRNA sequence.

Video S1. Gas-foaming process during 1 hour.

Video S2. Water contact of 3D NSs.

Video S3. Compression test of 3D NSs.

Video S4. Locomotor recovery of SCI rats at 8 wpi.
